# Upregulation of FAM83F by c-Myc promotes cervical cancer growth and aerobic glycolysis via Wnt/β-catenin signaling activation

**DOI:** 10.1038/s41419-023-06377-9

**Published:** 2023-12-16

**Authors:** Changlin Zhang, Lixiang Liu, Weizhao Li, Mengxiong Li, Xunzhi Zhang, Chi Zhang, Huan Yang, Jiayuan Xie, Wei Pan, Xue Guo, Peng She, Li Zhong, Tian Li

**Affiliations:** 1 Department of Gynecology, Pelvic Floor Disorders Center, Department of Orthopedics, Scientific Research Center, The Seventh Affiliated Hospital of Sun Yat-sen University, Shenzhen, China; 2 Shenzhen Key Laboratory of Chinese Medicine Active Substance Screening and Translational Research, Shenzhen, China; 3 College of Life Sciences and Oceanography, Shenzhen University, Shenzhen, China

**Keywords:** Cervical cancer, Targeted therapies

## Abstract

Cervical cancer (CC) seriously affects women’s health. Therefore, elucidation of the exact mechanisms and identification of novel therapeutic targets are urgently needed. In this study, we identified FAM83F, which was highly expressed in CC cells and tissues, as a potential target. Our clinical data revealed that FAM83F protein expression was markedly elevated in CC tissues and was positively correlated with poor prognosis. Moreover, we observed that FAM83F knockdown significantly inhibited cell proliferation, induced apoptosis, and suppressed glycolysis in CC cells, while its overexpression displayed opposite effects. Mechanistically, FAM83F regulated CC cell growth and glycolysis by the modulation of Wnt/β-catenin pathway. The enhancing effects of FAM83F overexpression on CC cell proliferation and glycolysis could be impaired by the Wnt/β-catenin inhibitor XAV939. Moreover, we found that c-Myc bound to the FAM83F promoter and activated the transcription of FAM83F. Notably, knockdown of FAM83F impaired the enhancement of cell proliferation and glycolysis induced by ectopic c-Myc. Consistent with in vitro findings, results from a xenograft mouse model confirmed the promoting role of FAM83F. In summary, our study demonstrated that FAM83F promoted CC growth and glycolysis through regulating the Wnt/β-catenin pathway, suggesting that FAM83F may be a potential molecular target for CC treatment.

**Figure:**
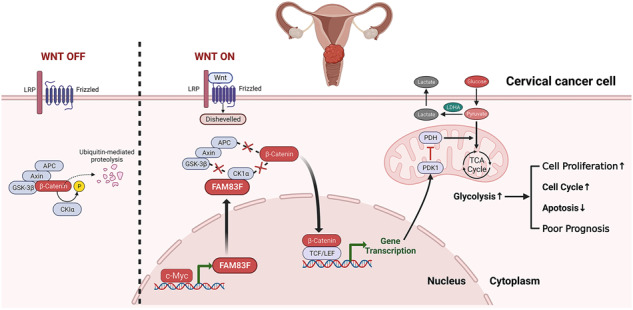
Schematic summary of c-Myc-activated FAM83F transcription to promote cervical cancer growth and glycolysis by targeting the Wnt/β-catenin signal pathway.

Schematic summary of c-Myc-activated FAM83F transcription to promote cervical cancer growth and glycolysis by targeting the Wnt/β-catenin signal pathway.

## Introduction

Cervical cancer (CC) is the fourth leading cause of cancer-related death and the fourth most common tumor among women globally, presenting a considerable burden to public health systems [1]. Most patients with early CC can achieve long-term survival after standard treatment. However, treatment for patients with advanced recurrent CC is difficult and ineffective, and the mortality rate is thus high [2, 3]. Gene targeting therapy and immunotherapy have become a hot research direction. Immune checkpoint blocking drugs targeting programmed cell death 1, programmed apoptosis ligand 1, and cytotoxic T lymphocyte antigen 4 are currently being tested for the treatment of recurrent/metastatic CC [4]. Unfortunately, the overall response rate to immune checkpoint blocking therapy is low (4–26%) [4]. Therefore, identifying potential key players in CC tumorigenesis for the development of novel cancer therapeutics is crucial.

The pathogenesis of CC involves various processes, including human papillomavirus (HPV) integration [5], microhomologous integration, histone methylation [6], epithelial–mesenchymal transition [7], and tumor microenvironment [8]. Recent research has mainly focused on ferroptosis [9], reactive oxygen species [10], autophagy [11], N6-methyladenosine modification [12, 13], glycolysis [14], and microRNAs [15]. Among them, cancer metabolism characterized by glycolysis has received more and more attention in cancer research [14, 16]. The Warburg effect, a hallmark of cancer, refers to the heavy reliance of cancer cells on glycolysis for energy, regardless of the presence of oxygen [17]. Sufficient evidence suggests that CC transfer is closely related to glycolysis. Recent research showed that N6-methyladenosine methyltransferase METTL3 promotes the proliferation and aerobic glycolysis of CC cells [13] and N6-methyladenosine regulates glycolysis in CC cells through PDK4 [12]. In addition, HPV E6/E7 proteins can promote aerobic glycolysis, proliferation, and metastasis in CC cells and promote CC progression by regulating *MYC* methylation sites [16]. Therefore, glycolysis-related molecules are potential targets for CC treatment, providing new ideas for further research on the pathogenesis and development of CC.

c-Myc is a transcription factor that regulates cell growth, differentiation, apoptosis, and metastasis [18], which is overexpressed in 50% of malignant tumor cells and is closely related to tumor invasiveness [19], drug resistance, and poor prognosis [20]. HPV integration is a key genetic event in CC transformation. Martin et al. revealed a potential integration mechanism mediated by microhomology and found that HPV integration into the flanking regions of the *MYC* gene induced c-Myc protein expression [7]. In 2022, the *MYC* family genes were identified as hot spots of HPV integration [5]. Besides, *Prevotella* overgrowth may contribute to the development of persistent HPV infection-associated cervical lesions by affecting c-Myc expression [21]. In tumor metabolism, c-Myc activates target genes encoding glycolytic enzymes and glucose transporters to drive glycolysis [22]. In addition, c-Myc can drive glycolysis and inhibit the thioredoxin-interacting protein [23, 24]. However, the specific mechanism of action of c-Myc in CC remains unclear. Here, we identified *FAM83F* as a potential target by screening public databases.

*FAM83F* is a member of the family of genes with sequence similarity 83 (*FAM83*) [25], which mediates oncogenic signaling in cancer and contains eight genes (*FAM83A–H*) [26]. The members of this family are characterized by the presence of a domain of unknown function 1669 in the N-terminus [27]. Recent studies have shown that FAM83F is a novel pro-oncogenic protein [28]. Moreover, FAM83F overexpression enhanced the migration of cells harboring mutant p53, demonstrating that it can activate mutant forms of p53 [25]. Another study showed that Circ-0000735 can promote glycolysis and inhibit cell apoptosis by regulating the expression of FAM83F [29]. To the best of our knowledge, no previous studies have investigated the correlation between FAM83F expression and CC. This study aimed to explore the function and mechanisms of action of FAM83F in CC.

In this study, we uncovered the unique function of FAM83F in CC and explored the potential molecular mechanisms. Our study is not only the first to reveal the role of FAM83F in CC progression but also identified a potential therapeutic target for CC treatment.

## Results

### High expression of FAM83F was associated with poor prognosis in patients with CC

**Fig. 1:**
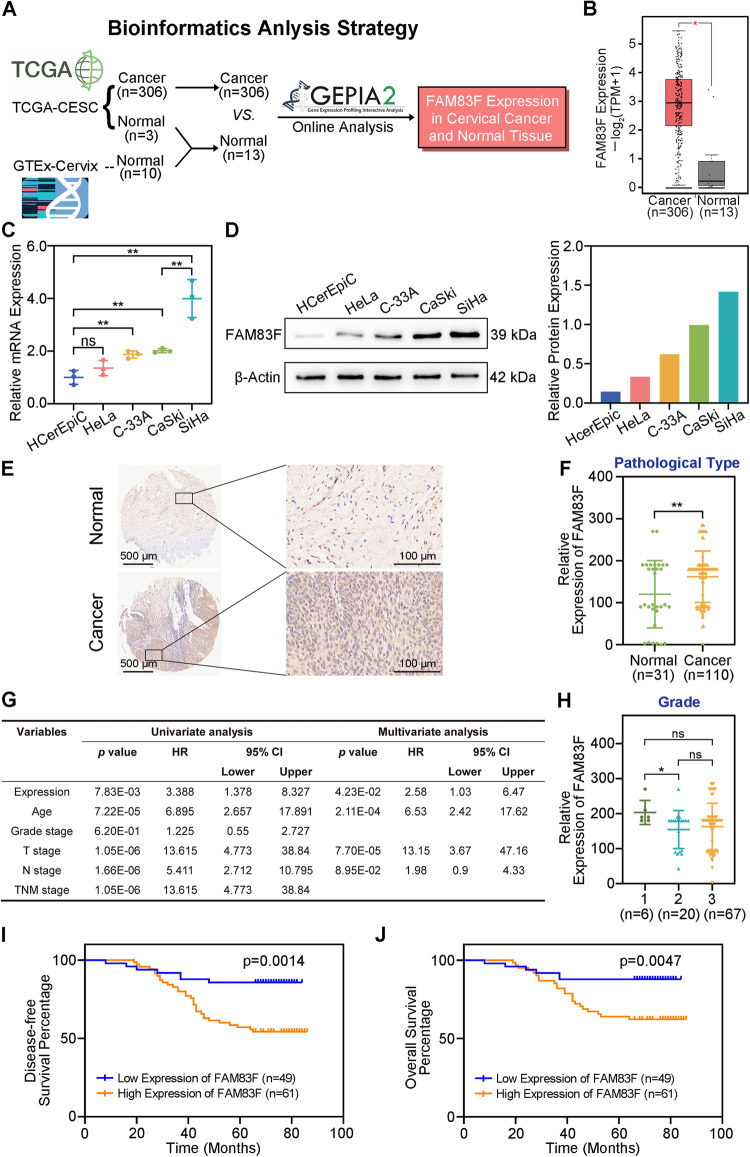
High expression of FAM83F was associated with poor prognosis in patients with CC. **A**, **B** Differences of FAM83F expression between CC and normal tissues were analyzed using GEPIA2 (http://gepia2.cancer-pku.cn/). **C**, **D** FAM83F was overexpressed in human CC cells. The mRNA and protein levels of FAM83F in several CC cell lines (HeLa, C-33A, CaSki, and SiHa) and normal cervical epithelial cells (HCerEpiC) were determined using qPCR and western blotting analyses. **E** The expression of FAM83F in para-carcinoma tissues and CC tumor tissues as determined using immunohistochemistry analysis. Scale bars, 500, 100 μm. **F** Mann–Whitney analyses of FAM83F protein expression in relation to pathological type. **G** Cox multivariate regression analyses of different expression levels of FAM83F protein and clinical characteristics of 110 patients with CC. **H** Mann–Whitney analyses of FAM83F protein expression in relation to grade. **I**, **J** Kaplan–Meier analysis showed poor disease-free survival and overall survival in patients with CC with high FAM83F expression. (*n* = 49 for low expression, *n* = 61 for high expression). The data represent the mean ± SD of three independent experiments, and the level of significance was indicated by ****P < 0.0001, ***P < 0.001, **P < 0.01, *P < 0.05. ns no significance (*p* > 0.05).

To investigate novel oncogenes involved in CC progression, we utilized Gene Expression Profiling Interactive Analysis 2 (GEPIA2) (http://gepia2.cancer-pku.cn/) to analyze the differences in FAM83F expression between CC tissue and normal tissue. Interestingly, we found that the mRNA level of FAM83F was significantly elevated in CC tissues compared with levels in normal tissues (*p* < 0.05, Fig. 1A, B).

Next, we analyzed FAM83F expression in normal cervical epithelial cells (HCerEpiC) and four CC cell lines (HeLa, C-33A, CaSki, and SiHa) using qPCR and western blotting. As shown in Fig. 1C, D, both mRNA and protein level of FAM83F were markedly up regulated in these CC cell lines compared to HCerEpiC. Furthermore, immunohistochemistry staining based on human CC tissue microarrays indicated a significant difference in the expression level of FAM83F between CC tissues and adjacent normal tissues (Fig. 1E, F).

The relationship between FAM83F and clinicopathological features of CC was evaluated next in clinical human CC tissue. The expression of FAM83F in tissues from 110 patients with CC was analyzed using immunohistochemistry and the protein expression levels were categorized as high and low. Multivariate regression analysis showed that the expression level of FAM83F, age, and T stage significantly correlated with CC prognosis (*p* < 0.05) (Fig. 1G). Additionally, regarding the relationship between FAM83F expression and clinicopathological variables in patients with CC, FAM83F expression was significantly associated with the tumor grade (Fig. 1H) but not with age, lymph node metastasis, and depth of invasion (Supplementary Fig. 1A–C).

The prognostic role of FAM83F in patients with CC was investigated using Kaplan–Meier analysis. High expression of FAM83F was significantly associated with decreased disease-free survival (*p* = 0.0014, Fig. 1I). Furthermore, overall survival analysis indicated that patients with low FAM83F expression had a significantly higher survival rate than those with high FAM83F expression (*p* = 0.0047, Fig. 1J). Collectively, these results indicate that FAM83F is significantly up-regulated in CC tissues and implicated in the progression of CC.

### FAM83F promoted cell proliferation, inhibited apoptosis, and accelerated cell cycle progression in CC cells

**Fig. 2:**
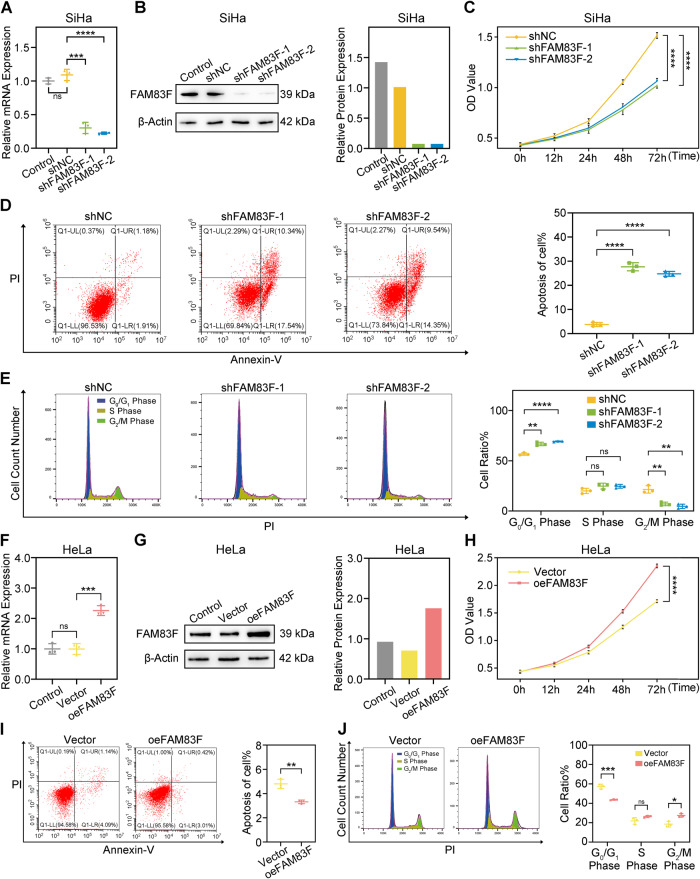
FAM83F promoted cell proliferation, inhibited apoptosis, and accelerated cell cycle progression in CC cells. **A**, **B** FAM83F was stably silenced in SiHa cells by transfection and selection. FAM83F expression was confirmed using qPCR and western blotting analysis. **C** CCK-8 assays indicated that knockdown of FAM83F inhibited the cell proliferation ability of SiHa cells. **D** The effect of FAM83F knockdown on cell apoptosis was determined in SiHa cells using flow cytometry. **E** Cell cycle distribution was determined in SiHa cells using flow cytometry analysis after FAM83F knockdown. The percentage of cells in the G_0_/G_1_, S, and G_2_/M phases was calculated. **F**, **G** FAM83F was overexpressed in HeLa cells by transfection and selection. FAM83F expression was confirmed using qPCR and western blotting analysis. **H** CCK-8 assays indicated that overexpression of FAM83F promoted the cell proliferation ability of HeLa cells. **I** The effect of FAM83F overexpression on cell apoptosis was determined in HeLa cells using flow cytometry. **J** Cell cycle distribution was determined in HeLa cells using flow cytometry analysis after FAM83F overexpression. The percentage of cells in the G_0_/G_1_, S, and G_2_/M phases was calculated. The data represent the mean ± SD of three independent experiments, and the level of significance was indicated by ****P < 0.0001, ***P < 0.001, **P < 0.01, *P < 0.05. ns no significance (*p* > 0.05).

To explore the biological function of FAM83F, we initially employed two independent short hairpin RNAs (shRNAs) to knocked down FAM83F expression in SiHa cells. The efficiency of FAM83F-targeting shRNAs was measured via qPCR and western blotting (Fig. 2A, B).

Next, we investigated the effects of FAM83F on CC cell growth. CCK-8 assay showed that FAM83F knockdown inhibited SiHa cell growth (Fig. 2C). Subsequently, flow cytometry analysis using Annexin-V/PI staining showed that the apoptosis rate was significantly higher in cells transfected with shFAM83F compared to cells transfected with shNC (Fig. 2D). Furthermore, we used flow cytometry to determine the role of FAM83F in the cell cycle. Compared to the shNC group, FAM83F silencing significantly raised the propagation of cells in the G_0_/G_1_ phase and reduced the propagation of cells in the G_2_/M phase (Fig. 2E). Consistently, FAM83F overexpression significantly promoted HeLa cell growth compared to the control group (Fig. 2F–H). Flow cytometry revealed that the effect of FAM83F overexpression on apoptosis (Fig. 2I) and cell cycle (Fig. 2J) in HeLa cells were opposed to those observed upon FAM83F knockdown in SiHa cells. Collectively, these results indicate that FAM83F plays a critical role in promoting cell proliferation and suppressing apoptosis in CC cells.

### FAM83F induces aerobic glycolysis to promote cell proliferation in CC cells

**Fig. 3:**
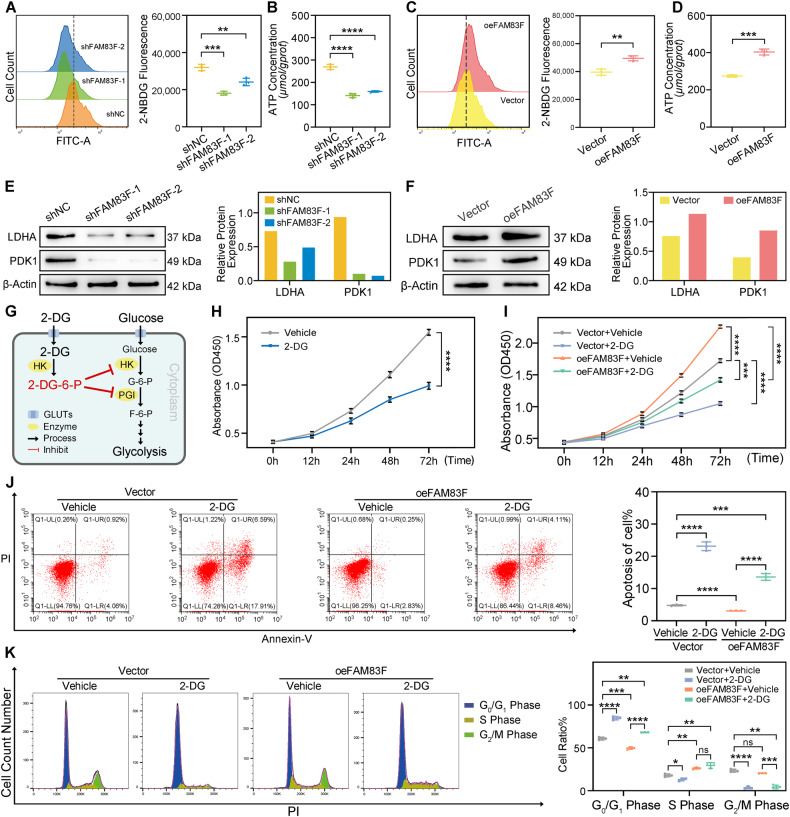
FAM83F induces aerobic glycolysis to promote cell proliferation in CC cells. **A**, **B** SiHa cells transfected with shNC, shFAM83F-1, or shFAM83F-2 were harvested to detect the levels of glucose uptake and ATP contents using specific kits. **C**, **D** HeLa cells that overexpressed FAM83F were harvested to detect the levels of glucose uptake and ATP contents using specific kits. **E**, **F** Western blotting was used to detect the protein levels of enzymes involved in glycolysis (LDHA and PDK1) in SiHa and HeLa cells, respectively, under the influence of FAM83F knockdown/overexpression. **G** Schematic diagram of the glycolysis inhibitor 2-deoxy-D-glucose (2-DG) (HK: hexokinase, PGI: Glucose 6-phosphate isomerase). **H**, **I** The proliferation curve of HeLa cells transfected with FAM83F overexpression plasmid or empty vector and treated with 2-DG or vehicle, as indicated. **J**, **K** The percentage of apoptotic cells (**J**) and cell cycle distribution (**K**) measured using flow cytometry of HeLa cells transfected as in (**I**). The data represent the mean ± SD of three independent experiments, and the level of significance was indicated by ****P < 0.0001, ***P < 0.001, **P < 0.01, *P < 0.05. ns no significance (*p* > 0.05).

Increased glycolysis has been coupled with various malignant phenotypes of CC cells, including tumor growth and metastasis [30]. Therefore, we investigated the impact of FAM83F on glycolysis in CC cells by measuring glucose uptake and ATP production after downregulation (Fig. 3A, B) or upregulation of FAM83F (Fig. 3C, D). Flow cytometry assays showed that FAM83F knockdown decreased glucose uptake in SiHa cells (Fig. 3A). In contrast, FAM83F overexpression boosted glucose uptake in HeLa cells (Fig. 3C). Since cancer cells predominantly generate energy from glycolysis, we also measured ATP production, an indicator of glycolysis. Our results revealed that FAM83F knockdown reduced ATP levels in SiHa cells compared to those in the control group (Fig. 3B), and FAM83F overexpression promoted ATP levels in HeLa cells (Fig. 3D). In addition, the expression of the glycolysis-related enzymes LDHA and PDK1 was measured using western blotting. Our results showed that FAM83F downregulation and overexpression separately decreased and increased the expression of glycolysis-related proteins LDHA and PDK1 in SiHa (Fig. 3E) and HeLa cells (Fig. 3F). In conclusion, FAM83F increased the glycolytic rate and promoted the Warburg effect in CC cells.

Next, we sought to determine whether FAM83F promoted cell proliferation through inducing glycolysis activation. HeLa cells were transfected with either a control or FAM83F overexpression vector, and then treated with control or the glycolysis inhibitor, 2-deoxy-D-glucose (2-DG) (Fig. 3G). As expected, 2-DG inhibited HeLa cell proliferation (Fig. 3H). After the addition of 2-DG, the CCK-8 assay results showed that 2‐DG strongly impeded the enhancement of cell proliferation induced by FAM83F overexpression in HeLa cells (Fig. 3I). Flow cytometry showed that 2-DG abolished FAM83F overexpression-mediated inhibition of apoptosis in HeLa cells (Fig. 3J). Besides, the FAM83F-mediated promotion of the cell cycle in HeLa cells was significantly impaired by 2-DG (Fig. 3K). Taken together, these results illustrate that FAM83F promotes cell proliferation and cell cycle progression and inhibits apoptosis by activating aerobic glycolysis.

### FAM83F regulated cell proliferation and glycolysis through the Wnt/β-catenin signaling pathway

**Fig. 4:**
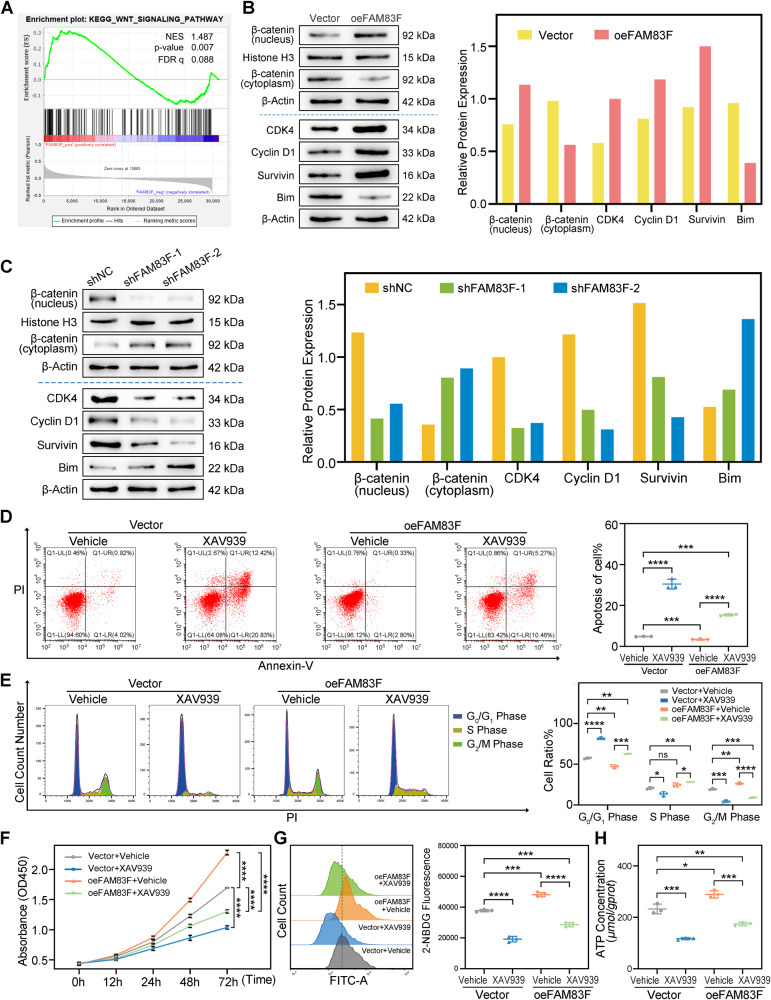
FAM83F regulated cell proliferation and glycolysis through the Wnt/β-catenin signaling pathway. **A** Gene Set Enrichment Analysis (GSEA) demonstrated that the Wnt/β-catenin signaling pathway was more correlated with patients with high FAM83F expression than patients with low FAM83F expression. NES, normalized enrichment score. **B**, **C** Western blotting was used to detect the protein levels of Wnt/β-catenin pathway-related factors and proapoptotic protein Bim under the influence of FAM83F overexpression or knockdown. FAM83F-overexpressing HeLa cells and their controls were treated in the absence or presence of the Wnt/β-catenin pathway inhibitor XAV939. Apoptosis (**D**), cell cycle progression (**E**), cell proliferation (**F**) and glycolysis capacity (**G**, **H**) were measured. The data represent the mean ± SD of three independent experiments, and the level of significance was indicated by ****P < 0.0001, ***P < 0.001, **P < 0.01, *P < 0.05. ns no significance (*p* > 0.05).

To explore the signaling pathways involved in FAM83F-mediated promotion of cell proliferation and glycolysis in CC cells, we conducted a pathway signal analysis. As indicated by Gene Set Enrichment Analysis (GSEA), FAM83F expression was strongly associated with the Wnt/β-catenin pathway (Fig. 4A), which is involved in cell growth, migration, invasion, and glycolysis [31, 32]. Hence, we performed western blotting to determine whether the Wnt/β-catenin pathway was activated in FAM83F-overexpressing CC cells. The protein levels of Wnt/β-catenin pathway-related factors (β-catenin in nucleus, CDK4, Cyclin D1, and survivin) were increased in FAM83F-overexpressing HeLa cells, while proapoptotic protein Bim was decreased (Fig. 4B). The opposite results were obtained in SiHa cells with stably knockdown of FAM83F (Fig. 4C). Taken together, these data suggest that FAM83F promotes the activation of the Wnt/β-catenin pathway in CC cells.

It has been well known the Wnt/β-catenin pathway is involved in aerobic glycolysis in tumor cells [33–35]. To explore whether the FAM83F regulates CC cell proliferation and glycolysis through Wnt/β-catenin pathway, HeLa cells that overexpressed FAM83F were treated with the Wnt/β-catenin inhibitor XAV939 in vitro. Flow cytometry showed that application of XAV939 reversed the effects of FAM83F overexpression-mediated inhibition of apoptosis (Fig. 4D) and promotion of the cell cycle progression (Fig. 4E) in HeLa cells. Consistently, as shown in Fig. 4F, the CCK-8 assays revealed that XAV939 treatment impaired the enhancement of cell proliferation induced by FAM83F overexpression in HeLa cells. Likewise, XAV939 blocked the increase of glucose uptake (Fig. 4G) and ATP production (Fig. 4H) in HeLa cells with stably overexpressing FAM83F. These results suggest that FAM83F enhances the capability of CC cells for proliferation and glycolysis by activating the Wnt/β-catenin pathway.

### c-Myc bound to the promoter of FAM83F to regulate its expression

**Fig. 5:**
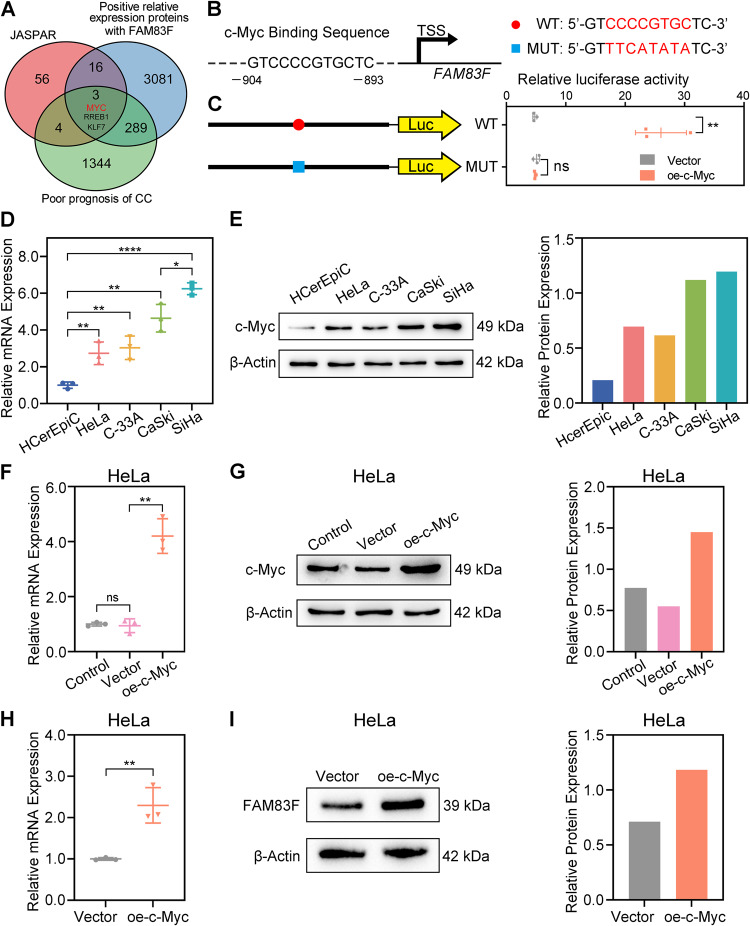
Transcription factor c-Myc bound to the FAM83F promoter to regulate FAM83F expression. **A** The transcription factor c-Myc bound to the promoter of FAM83F, as predicted by the JASPAR and TCGA database. **B** Binding site of c-Myc and FAM83F promoter sequence was predicted by the JASPAR database. **C** Relative luciferase activity of the FAM83F promoter was detected using a dual-luciferase reporter assay. **D**, **E** c-Myc expression was detected using qPCR and western blotting in CC cell lines (HeLa, C-33A, CaSki, and SiHa) and HCerEpiC. **F**–**I** The expression of c-Myc and FAM83F upon c-Myc overexpression in HeLa cells. The data represent the mean ± SD of three independent experiments, and the level of significance was indicated by ****P < 0.0001, ***P < 0.001, **P < 0.01, *P < 0.05. ns no significance (*p* > 0.05).

c-Myc plays an extremely important role in cell growth, metabolism, tissue development, and malignant transformation. Despite the importance of c-Myc in CC, evidence on the transcriptional regulation of FAM83F is unknown. We used JASPAR, a database of transcription factor binding profiles (genereg.net), to predict the FAM83F promoter. In addition, we identified proteins within TCGA database that were positively correlated with FAM83F expression and associated with poor prognosis in CC, respectively. After intersection, three candidate transcription factors were screened and c-Myc was selected (Fig. 5A). We next analyzed the FAM83F core promoter for a putative c-Myc-binding region using the JASPAR database of transcription factor-binding sites (Fig. 5B).

To determine whether these interactions were functionally significant, we performed a dual-luciferase assay and observed that the relative luciferase activity of the FAM83F wild-type promoter was higher in c-Myc-overexpressing HeLa cells than in cells transfected with a control vector (Fig. 5C). However, the relative luciferase activity of the cells transfected with mutated FAM83F promoter was similar in c-Myc-overexpressing and control vector-transfected HeLa cells (Fig. 5C). Furthermore, qPCR and western blotting indicated the mRNA (Fig. 5D) and protein expression (Fig. 5E) of c-Myc was increased in CC cell lines (HeLa, C-33A, CaSki, and SiHa) compared to HCerEpiC, a pattern that was consistent with FAM83F. Notably, the ectopic expression of c-Myc resulted in the transcriptionally up-regulation of FAM83F in HeLa cells (Fig. 5F–I). These findings indicate that c-Myc promotes FAM83F transcription by binding to the promoter of FAM83F.

### c-Myc promotes cell proliferation and glycolysis via upregulating FAM83F in CC cells

**Fig. 6:**
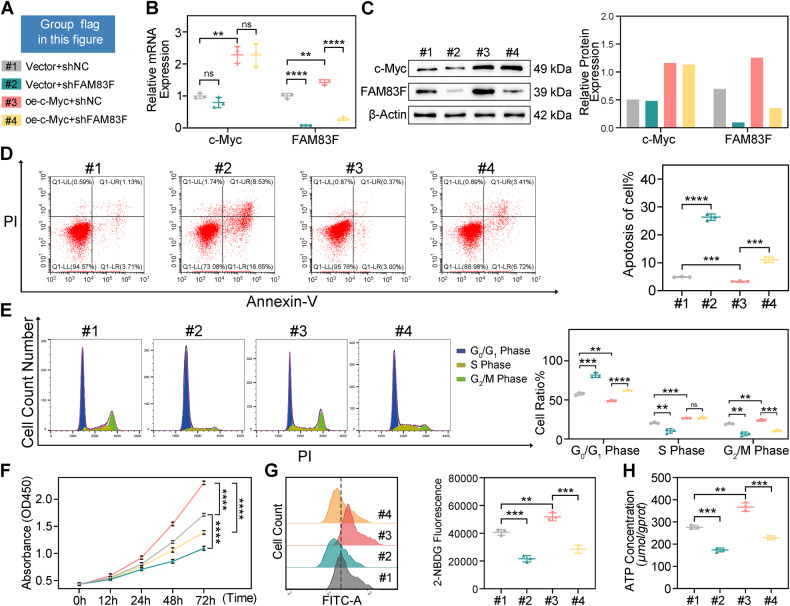
c-Myc promotes cell proliferation and glycolysis via up-regulating FAM83F in CC. **A** Group flag in this figure. HeLa cells were transfected with FAM83F shNC or specific shRNAs and control vector or c-Myc overexpression plasmids. **B**, **C** The expression of c-Myc and FAM83F detected using qPCR and western blotting. **D** Flow cytometry to evaluate the apoptosis rate and cell cycle distribution of HeLa cells. **E** Flow cytometry to evaluate the cell cycle of HeLa cells. **F** Proliferation of HeLa cells using a CCK-8 assay. **G**–**H** Glucose uptake and ATP contents were assessed. The data represent the mean ± SD of three independent experiments, and the level of significance was indicated by ****P < 0.0001, ***P < 0.001, **P < 0.01, *P < 0.05. ns no significance (*p* > 0.05).

We showed that c-Myc regulates FAM83F expression by binding to its promoter. Next, we sought to determine whether c-Myc promotes CC cell proliferation and glycolysis by regulating FAM83F. As illustrated in Fig. 6A–C, qPCR (Fig. 6B) and western blotting (Fig. 6C) showed that overexpression of c-Myc promoted the expression of FAM83F in HeLa cells. Besides, the significant reduction in cell apoptosis (Fig. 6D) and cell cycle arrest (Fig. 6E) induced by c-Myc overexpression was partially recovered by FAM83F knockdown in HeLa cells. The proliferation of HeLa cells notably promoted by the overexpression of c-Myc was reversed by the silencing of FAM83F expression (Fig. 6F). Consistent with these findings, overexpression of c-Myc increased glucose uptake (Fig. 6G) and ATP production (Fig. 6H) compared to vector-transfected cells, whereas the enhancement in glycolysis was attenuated after cotransfection with shFAM83F. Collectively, these data suggest that c-Myc promotes cell proliferation and glycolysis by upregulating FAM83F expression in CC cells.

### Intervention on the c-Myc/FAM83F/Wnt/β-catenin axis inhibited CC progression in a mouse xenograft model

**Fig. 7:**
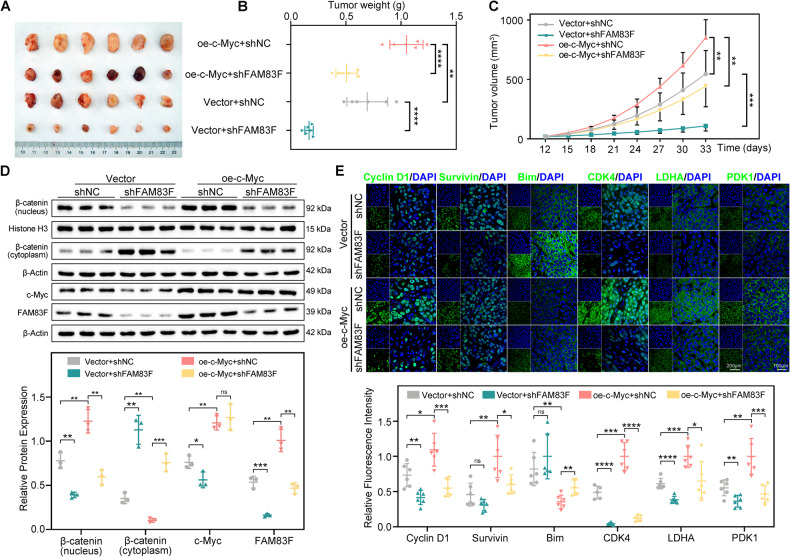
Intervention in the c-Myc/FAM83F/Wnt/β-catenin axis inhibited CC progression in a mouse xenograft model. **A** Morphology of tumor xenograft from each mouse (*n* = 6 for each group). **B** Tumor weight from each mouse at the end of the experiment. **C** Tumor volume from each mouse was measured and recorded every three days during the experiment. **D** The expression of c-Myc, FAM83F, β-catenin in the nucleus, and β-catenin in cytoplasm in tumor xenografts were assessed using western blotting. **E** Immunofluorescence was used to detect the protein levels of Wnt/β-catenin pathway-related factors (Cyclin D1, survivin, and CDK4), proapoptotic protein Bim, and enzymes involved in glycolysis (LDHA and PDK1). Scale bars, 200 μm and 100 μm. The data represent the mean ± SD of three independent experiments, and the level of significance was indicated by ****P < 0.0001, ***P < 0.001, **P < 0.01, *P < 0.05. ns no significance (*p* > 0.05).

To further confirm the oncogenic role of FAM83F in CC progression and its dependence on the transcription factor c-Myc and Wnt/β-catenin signaling pathway in vivo, we constructed CC xenograft mouse models with or without c-Myc overexpression or FAM83F knockdown. The animals were randomly divided into four groups (six per group): vector + shNC, vector + shFAM83F, oe-c-Myc + shNC, and oe-c-Myc + shFAM83F. Tumor weights and volumes were measured. c-Myc overexpression promoted tumor growth in size (Fig. 7A), weight (Fig. 7B), and volume (Fig. 7C), whereas FAM83F knockdown markedly suppressed tumor growth. Furthermore, FAM83F knockdown markedly reversed the growth promotion induced by c-Myc overexpression. In addition, no other signs of acute or delayed toxicity were observed in the mice during treatment.

Furthermore, western blotting analysis of xenograft tumor tissue lysates demonstrated that knockdown of FAM83F significantly impeded the promotion of β-catenin nuclear accumulation induced by c-Myc overexpression (Fig. 7D). Moreover, the expression of c-Myc, FAM83F, and Wnt/β-catenin pathway-related factors (Cyclin D1, survivin, and CDK4), proapoptotic protein Bim and glycolysis-related proteins (LDHA and PDK1) were determined using immunofluorescence staining (Fig. 7E and Supplementary Fig. 2). Consistent with the in vitro results, FAM83F knockdown significantly impaired the elevated protein levels of Wnt/β-catenin pathway-related factors and glycolysis-related proteins induced by c-Myc overexpression. These results from the xenograft model indicate that FAM83F knockdown has an anti-cancer effect in CC by inhibiting the Wnt/β-catenin pathway and the effect of c-Myc overexpression was reversed by FAM83F knockdown. Our proposed model in graphical abstract illustrates the function of the c-Myc/FAM83F/Wnt/β-catenin axis in CC progression.

## Discussion

CC is one of the deadliest malignant tumors of the female reproductive system worldwide [36, 37]. Although the treatment of CC has advanced in the previous decades [38, 39], metastasis and recurrence of CC continue to pose challenges to physicians and patients [2]. Therefore, identifying new targets for the development of novel cancer therapeutics is important.

FAM83F is involved in numerous cancers [40, 41]. For instance, FAM83F overexpression suppresses the proliferation, migration, invasion, and glycolysis of NSCLC cells [40]. Moreover, FAM83F regulates the biology and differentiation of thyroid follicular cells by cross-regulating the MAPK and TGF-β signaling pathways [28]. High expression of FAM83F in breast cancer tissues has adverse effects on the survival outcome of patients, rendering FAM83F a potential target for the clinical treatment of breast cancer [42]. However, the role of FAM83F in CC mobility and its relationship to tumor glycolysis have not been clarified. In this study, we provide evidence that FAM83F may serve as a potential prognostic biomarker or therapeutic target in CC. FAM83F was highly expressed in CC cell lines and CC tissues. High expression of FAM83F was correlated with poor prognosis of patients with CC. In addition, our results revealed that inhibition of endogenous FAM83F expression suppressed cell growth, induced apoptosis, and suppressed glycolysis in vitro. Moreover, we observed that tumorigenesis was suppressed in xenografts of CC cells wherein FAM83F was silenced, indicating that FAM83F may function as an essential regulator in CC. To the best of our knowledge, this is the first study to determine the role of FAM83F in the development and progression of CC.

Glycolysis has been associated with CC cell proliferation and apoptosis [43, 44]. Interestingly, glycolysis was related to the expression of FAM83F [29]. Our study showed that FAM83F overexpression promoted cell proliferation and glycolysis in CC cells. Moreover, treatment with the glycolytic inhibitor 2-DG [45] greatly reduced the capacity of FAM83F overexpression to promote proliferation and glycolysis, which shows that FAM83F promotes CC cell proliferation by activating glycolysis. Upregulation of myosin 1b expression has been shown to promote glycolysis, migration, and invasion of CC cells by stimulating downstream glycolysis-related genes [43]. Knockdown of CircCDK17 repressed cell proliferation, migration, invasion, and glycolysis, and promoted cell apoptosis in CC [46]. In our study, the role of FAM83F in the migration and metastasis of CC cells was not tested, which is a limitation.

FAM83F is a mediator of the typical Wnt/β-catenin signaling pathway [40], which has been widely implicated as a controller of cell growth, migration, and stem-like phenotype [47–49]. Karen Dunbar et al. demonstrated that FAM83F regulates wnt signaling through an interaction with CK1α at the plasma membrane [50]. Inactivation of the Wnt/β-catenin pathway occurs via inhibition of the nuclear transfer of β-catenin by CC cells [51]. Our GSEA revealed that FAM83F expression was strongly associated with the Wnt/β-catenin pathway. Interestingly, Zhang et al. found that DEPDC1 promotes aerobic glycolysis through the Wnt/β-catenin pathway [52]. MIR-G-1 promotes serum starvation-induced nuclear macrophage/autophagy and accelerates paclitaxel-induced DNA damage repair in CC cells by mediating the activation of the Wnt/β-catenin pathway [53]. Consistently, we found that the expression of Wnt/β-catenin pathway-related factors significantly decreased after FAM83F knockdown. Furthermore, the promoting effects of FAM83F overexpression on CC proliferation and glycolysis were impaired by the Wnt/β-catenin inhibitor XAV939 [54]. Future research may reveal whether other pathways are also involved in FAM83F-mediated growth of CC cells.

Overexpression of c-Myc can promote glycolysis [18, 55], thereby increasing the energy source of cancer cells and promoting their proliferation, migration, and invasion; however, the specific mechanism remains to be elucidated. Hexokinase 2 (*HK2*) and glucose transporter-1 (*GLUT1*) serve as downstream genes of c-Myc [22]. Therefore, downregulation of c-Myc expression may promote cancer cell apoptosis by inhibiting the glycolytic pathway, making c-Myc a potential target for glycolysis-related therapy in tumors [56]. Other studies have shown that the expression of c-Myc and FAM83F is upregulated in lung adenocarcinoma [57]. However, whether c-Myc binds to the FAM83F promoter to regulate the Warburg effect in CC was unknown. We found that c-Myc binds to the FAM83F promoter region and that c-Myc overexpression promotes the Warburg effect and CC cell proliferation. Interestingly, the Wnt/β-catenin pathway is an activator of c-Myc. However, the precise mechanism behind c-Myc activation in CC remains largely unknown and whether the FAM83F/Wnt/β-catenin axis facilitates CC progression in a c-Myc-dependent positive feedback loop needs to be studied in the future. Lastly, although FAM83F knockdown inhibited CC progression in a mouse xenograft model, we did not verify the role of FAM83F in tumor metastasis in animal experiments. The above issues remain the direction and goal of our future studies, and our efforts will focus on designing appropriate experiments.

High expression of FAM83F is significantly correlated with poor prognosis in patients with lung cancer [58], thyroid papillary carcinoma [28], and breast cancer [42]. In this study, we evaluated the relationship between FAM83F expression and clinical prognosis and found, for the first time, that high FAM83F expression in patients with CC predicted poor prognosis.

In summary, our study revealed a potential oncogenic role for FAM83F in CC. Our results demonstrate that c-Myc binds to the FAM83F promoter to activate its expression, thus promoting CC growth through glycolysis via the Wnt/β-catenin pathway in vitro and in vivo. Furthermore, high expression of FAM83F was associated with poor prognosis in patients with CC, suggesting that FAM83F could be a potential biomarker for the diagnosis and a therapeutic target for CC in the future.

## Materials and methods

### Gene expression data source

The CC dataset was obtained from GTEx (https://www.genome.gov) and the Cancer Genome Atlas project (TCGA, https://tcga-data.nci.nih.gov/tcga/). Differences of FAM83F expression between CC tissue and normal tissue were analyzed by Gene Expression Profiling Interactive Analysis 2 (GEPIA2)(http://gepia2.cancer-pku.cn/).

### Gene set enrichment analysis (GSEA)

The GSEA software tool (version 2.0.13, www.broadinstitute.org/gsea/) was used to identify KEGG pathways (MSigDB, version 4.0) that show an overrepresentation of up- or downregulated genes between FAM83F high expression and low expression (1645434177503, 1645434193922). Briefly, an enrichment score was calculated for each gene set (i.e., KEGG pathway) by ranking each gene by their expression difference using Kolmogorov-Smirnov statistic, computing a cumulative sum of each ranked in each gene set, and recording the maximum deviation from zero as the enrichment score.

### Cell lines and cell culture

Normal cervical epithelial cells (HcerEpic) were purchased from ATCC, and cultured in Cervical Epithelial Cell Basal Medium (PCS-480-032, ATCC, USA), added with growth factors (PCS-480-042, ATCC, USA). Four cervical cancer cell lines (HeLa, C-33A, Caski and SiHa), were from ATCC, authenticated by short tandem repeat profiling and cultured in DMEM with 10% FBS, 100 μg/ml streptomycin and 100 U/ml penicillin. All cells were maintained in standard culture condition and tested negative for Mycoplasma using MycoBlue Mycoplasma Detector Kit (D101-02, Vazyme, China).

### Reagents and antibodies

The antibodies below are all purchased from abcam: anti-FAM83F (ab272651), anti-β-catenin (ab32572), anti-c-Myc (ab32072), anti-CDK4 (ab108357), anti-Cyclin D1 (ab16663), anti-Survivin (ab76424), anti-Bim (ab32158), anti-LDHA (ab52488), anti-PDK1 (ab202468), anti-Histone H3(ab1791) and anti-β-actin (ab8226). All antibodies are used as the product datasheet. Goat anti-rabbit IgG (H&L) secondary antibody (Alexa Flour 488® conjugate, ab150077) and goat anti-mouse IgG (H&L) secondary antibody (Alexa Flour ®488 conjugate, ab150113) are also purchased from abcam. The HRP-conjugated secondary antibody against mouse and rabbit are purchased from Proteintech (SA00001-1, SA00001-2). Quantification and analysis were performed using Image J software.

### Real-time PCR (qPCR)

Total RNA was isolated using RNA-Quick Purification Kit (RN001, ES Science, China), and cDNA was synthesized using the Fast All-in-One RT Kit (RT001, ES Science, China). The SYBR Green PCR master mix (Q712, Vazyme, China) was then used for qPCR, which was followed by detection with a Bio-Rad CFX96 and analyzed with the Bio-Rad Manager software (Bio-Rad, Hercules, CA) (see Table S1 for sequences). Each sample was tested in triplicate, respectively.

### Western blot

Whole cell lysates or nuclear extracts were prepared using Complete Lysis-M reagent (Roche, Indianapolis, IN) and RIPA lysis buffer (Beyotime Biotechnology, Shanghai, China). Protein concentration was determined by BCA assay (ThermoFisher Scientific, Waltham, MA). The proteins were separated in 10% SDS-PAGE gels and transferred onto PVDF membranes for detection (see Supplemental Material for original western blots).

### Cell counting kit-8 (CCK-8) assay

CCK-8 (K1018, apexbio, China) was applied to determine cell proliferation levels. Transfected cells at a density of 5.0 × 10^3^ cells per well were seeded in a 96-well plate and then cultured for 12, 24, 48, or 72 h. CCK-8 reagent was applied according to the instruction manual. The proliferation levels were determined by measuring the absorbance at 450 nm using a Multifunctional microplate reader (Synergy H1M, Biotek, USA).

### Apoptosis and cell cycle assay

For apoptosis detection, cells (1 × 10^5^ cells/ml) were stained with AnnexinV-FITC and PI according to the manufacturer’s instructions (40302ES50, Yeasen, Shanghai), and then analyzed by flow cytometry (CytoFLEX, Beckman Coulter, Brea, CA). For cell cycle detection, cells (1×10^5^ cells/ml) were stained with PI and analyzed by flow cytometry using the kit and machine as apoptosis detection. Each group was tested in triplicate, respectively.

### Metabolic assay

Glucose uptake of cells was measured using commercial kits (2-NBDG Glucose Uptake Assay Kit (Cell-Based), #K682-50, BioVision, USA) following the manufacturer’s instructions. According to the manufacturer’s instructions, ATP was quantified by using Cell Titer-Glo® luminescence assay (Promega). All samples were tested in triplicate, respectively.

### Plasmids construction and transfection

*FAM83F* shRNA sequences were cloned into the pLKO.1 vector to obtain pLKO.1-shFAM83F-1/2 plasmid (see Table S1 for sequences). The *FAM83F* and *MYC* sequences were synthesized and cloned into the pLVX-puro vector to obtain the overexpression plasmid. For transfection, HEK293T cells were transfected with plasmid and virus of it was collected at 48 h and 72 h. The supernantant-containing virus infected SiHa or HeLa cells with polybrane. The transfected cells were selected with puromycin (2 μg/ml) more than 1 week.

### Dual-luciferase reporter assay

Using the promoter sequence of FAM83F as a query, we searched for c-Myc binding sites by using JASPAR software (http://jaspar.genereg. net/). Dual-luciferase reporter assay was used to detect the interaction between c-Myc and FAM83F promoter region. FAM83F promoter fragments were cloned into the pGL3 Basic vector. The wild-type FAM83F promoter luciferase (Luc) and mutant FAM83F promoter luciferase were constructed by GenePharma. Transfections were carried out in HeLa cells by using Lipofectamine™2000 transfection reagent (Invitrogen) according to the manufacturer’s suggestion. After 36 h, cells were lysed with passive lysis buffer using the luciferase kit (Promega, Madison, WI, USA). The reporter activities were determined using the Dual-Glo Luciferase Assay system (Promega, Madison, WI, USA). The ratio of Firefly: Renilla luciferase was calculated and the results presented as relative luciferase activity.

### Confocal immunofluorescence assay

For confocal microscopy analysis, cells grown on chamber slides were washed with PBS, fixed with paraformaldehyde, and permeabilized with pre-cooled methyl alcohol for 15 min. The samples were then pretreated with 10% bovine serum albumin (BSA) in PBS for 30 min, and specific antibodies were added and incubated overnight at 4 °C. Following five times of 5 min washes with PBS, secondary antibodies were added and incubated for another hour. After five times of additional 5 min washes, samples were examined using the confocal microscope (LSM 880 With Airyscan, Zeiss, Germany).

### Xenograft experiments

Four- to six-week-old nude mice were acquired from Hangzhou Ziyuan Experimental Animal Technology Co., LTD (Hangzhou, China) and kept in a SPF animal facility. All animal experiments were conducted according to the institutional ethical and safe guidelines (L102012023020E, Institutional Animal Welfare and Ethics Committee, Sun Yat-sen University Cancer Center, Guangzhou, China). No statistical methods were used to predetermine the sample size. No blinding method was used during the experimental procedure. There were no animal exclusion criteria. The animals were randomly divided into four groups (six per group) and HeLa cells with stably transfected vector + shNC, vector + shFAM83F, oe-c-Myc + shFAM83F or oe-c-Myc + shNC were subcutaneously injected into the nude mice (5 × 10^6^ cells per mouse). Tumor length and width were measured every 3 days until the experiment was ended. The tumor size was measured using a Vernier caliper, and the tumor volume was calculated as *V* = (length × width × width)/2. At the end of the experiment, the mice were sacrificed, and the tumors were removed and examined.

### CC tissue microarray and immunohistochemistry (IHC)

The CC tissue microarray (HUteS154Su01) for evaluating FAM83F expression was purchased from Shanghai Outdo Biological Technology Co. LTD (Shanghai, China). Clinicopathologic information was documented for all cases. IHC was performed in accordance with the arrays stained with indicated antibodies, followed by counterstaining with standard protocols. The slides were scanned, and the images were then digitalized for quantitative evaluation. The integrated optical density was analyzed using an immunoreactive score.

### Statistical analysis

Each experiment was repeated at least three times and the results were presented as the mean ± SD. Our calculations were done with SPSS (Version 20.0, Abbott Laboratories, USA) and GraphPad Prism 9. The student’s *t*-test was used for comparisons between two groups and one-way ANOVA was used for comparisons among multiple groups. The variance was similar between the groups that were being statistically compared. The survival probability was calculated using the Kaplan-Meier method. *P < 0.05, **P < 0.01, ***P < 0.001 and ****P < 0.0001. ns, no significance.

### Supplementary information

Supplementary figure legends

Supplementary Figure 1

Supplementary Figure 2

Supplementary Table 1

Reproducibility checklist

Original Data File

## Data Availability

All data needed to evaluate the conclusions in the paper are present in the paper and the Supplementary information. Additional data related to this paper may be requested from the authors.
