# Supplementary material for: Upregulation of FAM83F by c-Myc promotes cervical cancer growth and aerobic glycolysis via Wnt/β-catenin signaling activation
Source: Cell Death Dis. 2023 Dec 16;14(12):837. doi: 10.1038/s41419-023-06377-9 (PMC10725447; doi:10.1038/s41419-023-06377-9)
Supplement: Supplementary file 1 — Supplementary figure legends [file 41419_2023_6377_MOESM1_ESM.docx]

**Supplementary figure legends：**

**Supplementary Figure 1. Expression of FAM83F in cervical tissue of CC and its relationship with clinical data**

**A–C** Mann–Whitney analyses of FAM83F protein expression in relation with age, T stage and N stage.

**Supplementary Figure 2. Overexpression of c-Myc recovered the expression of c-Myc and FAM83F inhibited by FAM83F knockdown in a mouse xenograft model**

**A, B** Immunofluorescence was used to detect the protein levels of c-Myc and FAM83F. Scale bars, 200μm,100 μm.
