# Supplementary figures and images for: Upregulation of FAM83F by c-Myc promotes cervical cancer growth and aerobic glycolysis via Wnt/β-catenin signaling activation
Source: Cell Death Dis. 2023 Dec 16;14(12):837. doi: 10.1038/s41419-023-06377-9 (PMC10725447; doi:10.1038/s41419-023-06377-9)

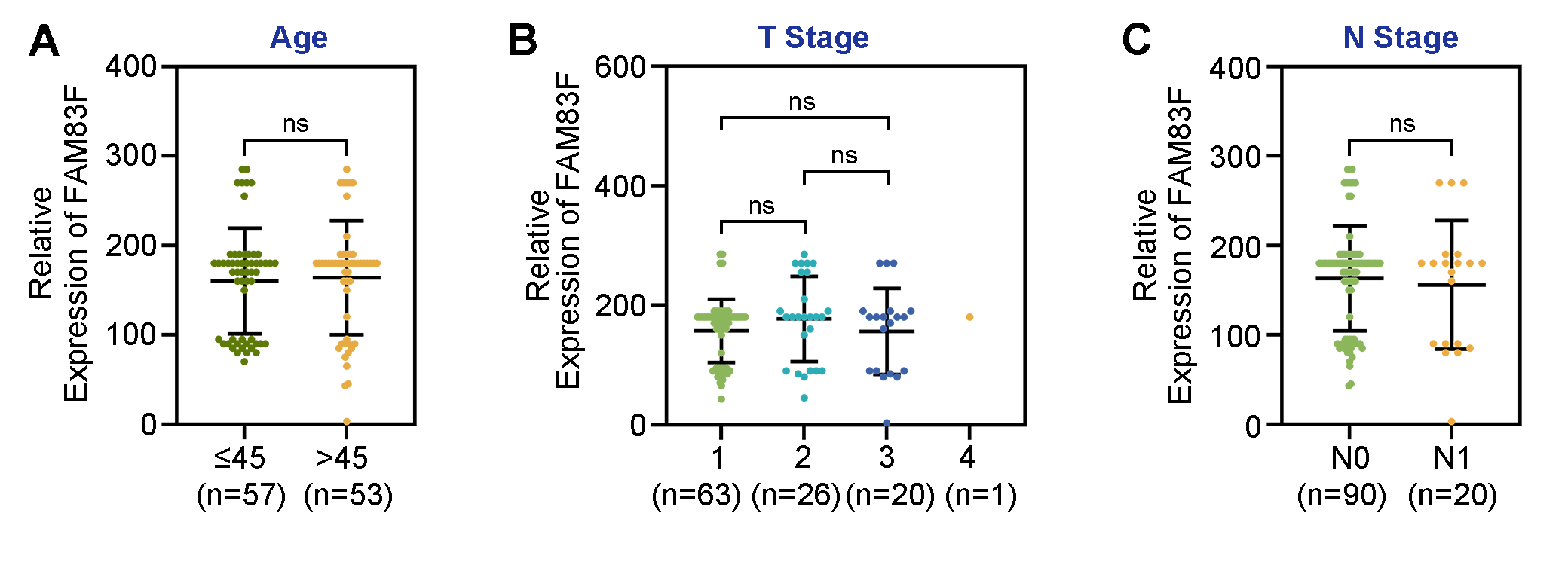

Supplement: Supplementary file 2 — Supplementary Figure 1 [file 41419_2023_6377_MOESM2_ESM.tif]

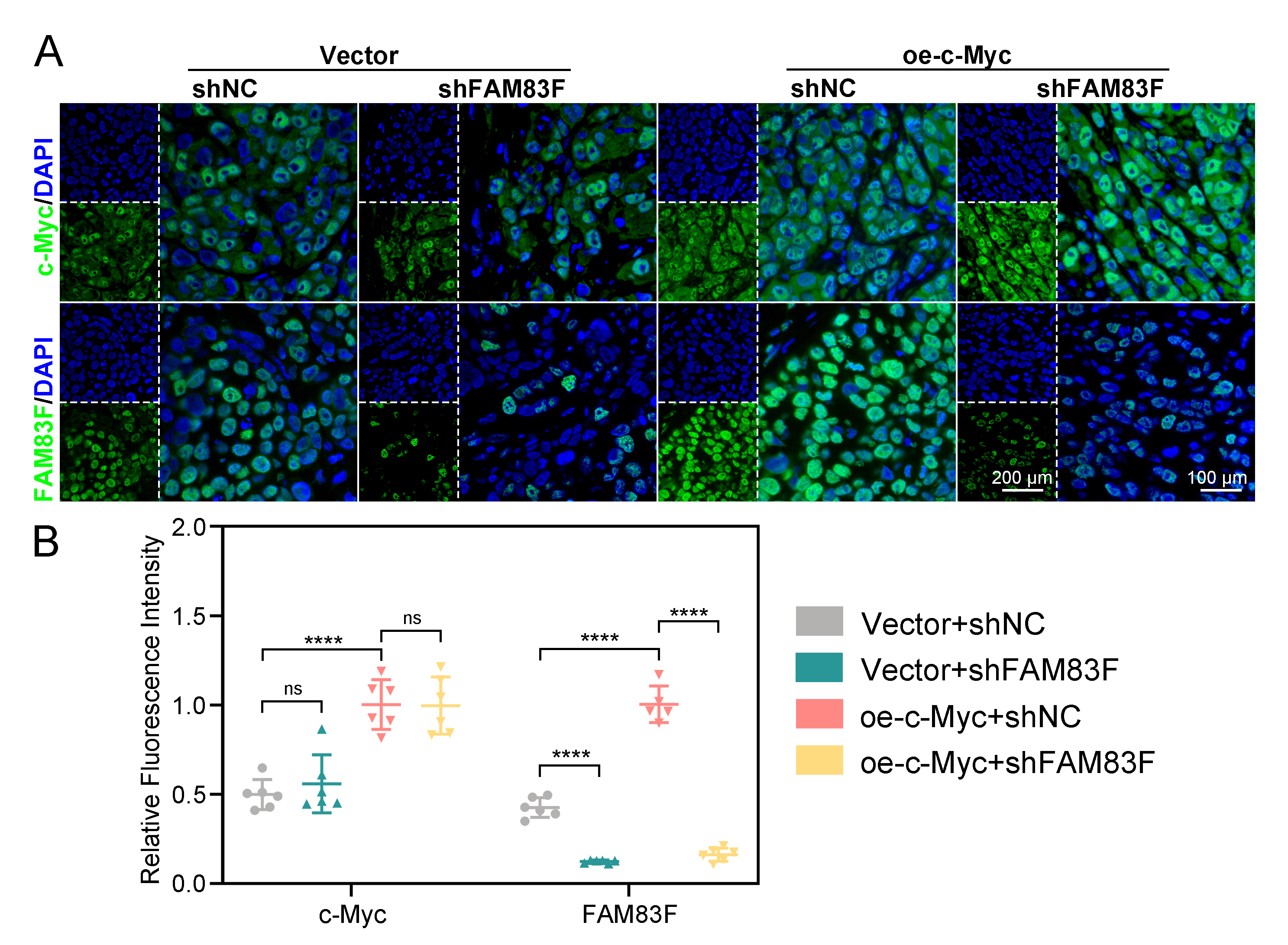

Supplement: Supplementary file 3 — Supplementary Figure 2 [file 41419_2023_6377_MOESM3_ESM.tif]
