# Supplementary material for: Upregulation of FAM83F by c-Myc promotes cervical cancer growth and aerobic glycolysis via Wnt/β-catenin signaling activation
Source: Cell Death Dis. 2023 Dec 16;14(12):837. doi: 10.1038/s41419-023-06377-9 (PMC10725447; doi:10.1038/s41419-023-06377-9)
Supplement: Supplementary file 4 — Supplementary Table 1 [file 41419_2023_6377_MOESM4_ESM.docx]

**Supplementary Table 1: shRNA and PCR Primer Sequences**

|  | Genes | Forward primer (5′→3′) | Reverse primer (5′→3′) |
| --- | --- | --- | --- |
| qPCR | MYC | 5’-GGTAGTGGAAAACCAGCAGCCT-3’ | 5’-GTGGGCAGCAGCTCGAATTT-3’ |
|  | FAM83F | 5’-GATGGTGGACGGTGACAAAG-3’ | 5’-CAGTGGAGGAGTAATGGAGGC-3’ |
|  | β-actin | 5’-CCTTCCTTCCTGGGCATGG-3’ | 5’-GATCTTCATTGTGCTGGGTGC-3’ |
| knockdown | shFAM83F-1 | 5’-CCGGTCCGATGGTGGACGGTGACAAATTCAAGAGATTTGTCACCGTCCACCATCTTTTTGGAAG-3’ | 5’-AATTCTTCCAAAAAGATGGTGGACGGTGACAAATCTCTTGAATTTGTCACCGTCCACCATCGGA-3’ |
|  | shFAM83F-2 | 5’-CCGGTCCGGTGATATCTTTCAAGACATTCAAGAGATGTCTTGAAAGATATCACCTTTTTGGAAG-3’ | 5’-AATTCTTCCAAAAAGGTGATATCTTTCAAGACATCTCTTGAATGTCTTGAAAGATATCACCGGA-3’ |
|  |  |  |  |
