# Supplementary material for: Upregulation of FAM83F by c-Myc promotes cervical cancer growth and aerobic glycolysis via Wnt/β-catenin signaling activation
Source: Cell Death Dis. 2023 Dec 16;14(12):837. doi: 10.1038/s41419-023-06377-9 (PMC10725447; doi:10.1038/s41419-023-06377-9)

Original western blots

Relevant areas for cropped blots in the main and Extended Data figures are shown with a dashed box.

Figure 1D

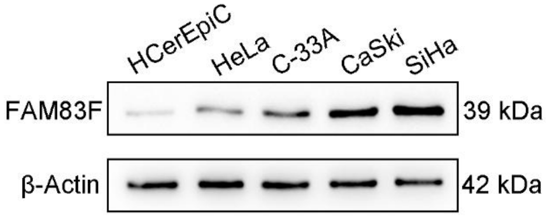

FAM83F 39kDa

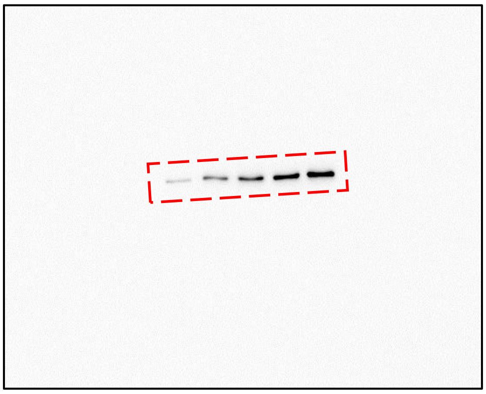

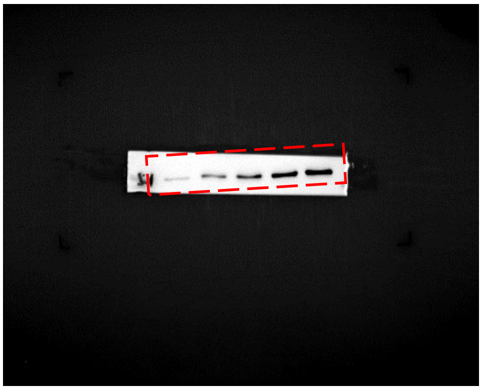

β-Actin 42kDa

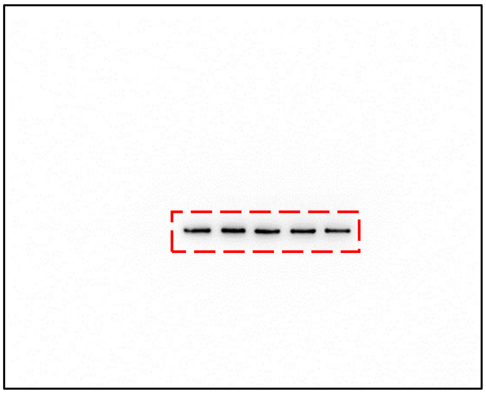

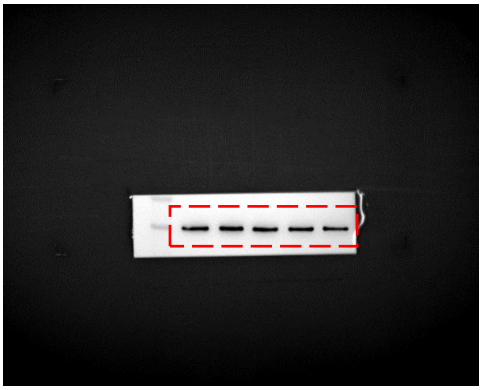

Figure 2B

B

SiHa

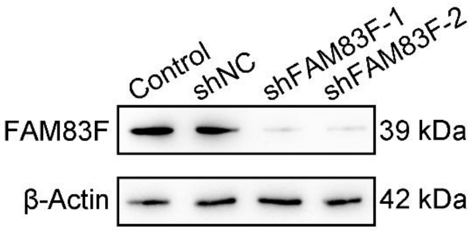

FAM83F 39kDa

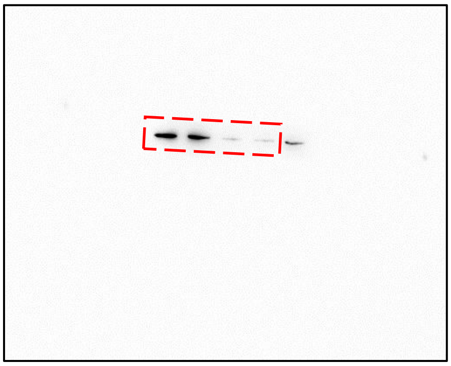

β-Actin 42kDa

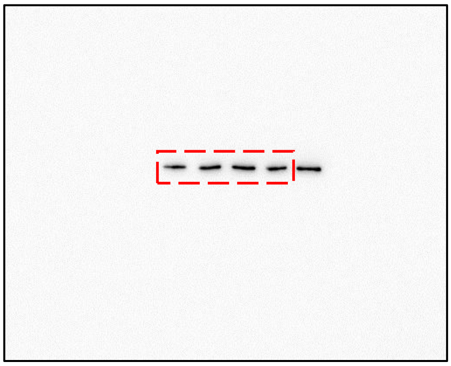

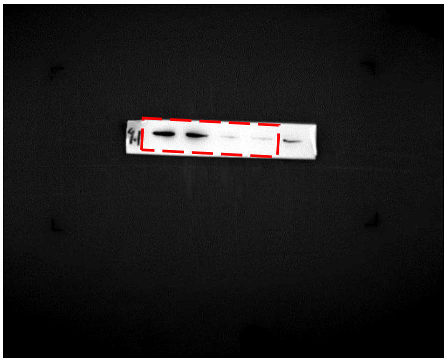

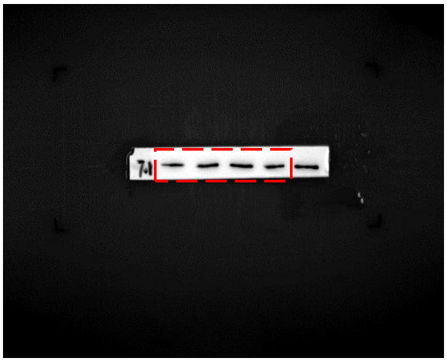

Figure 2G

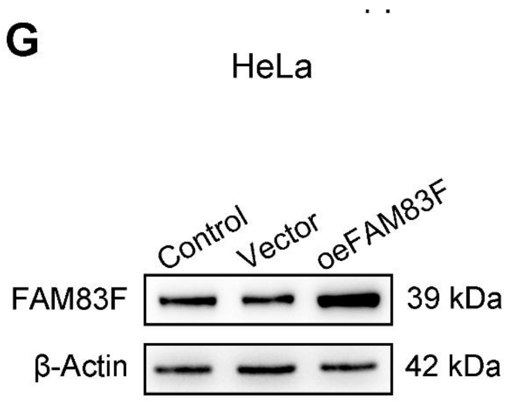

FAM83F 39kDa

β-Actin 42kDa

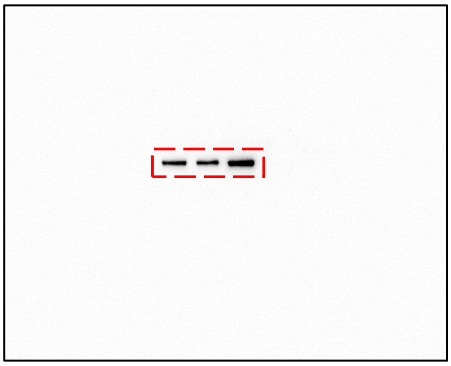

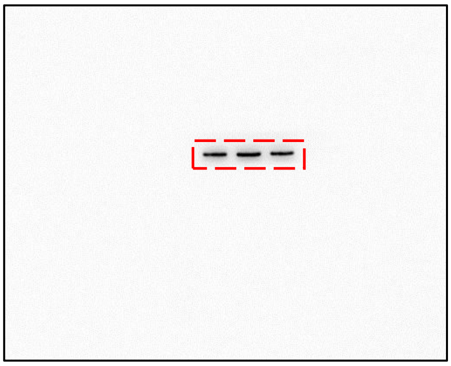

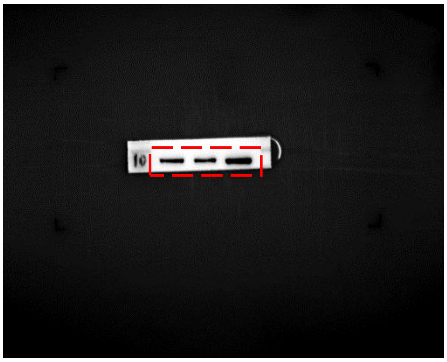

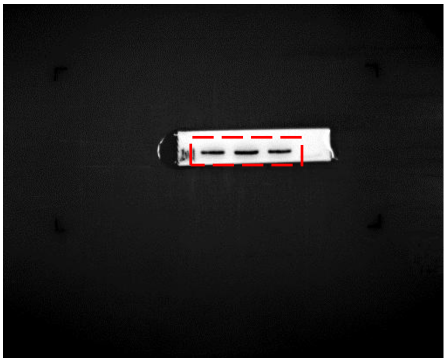

Figure 3E

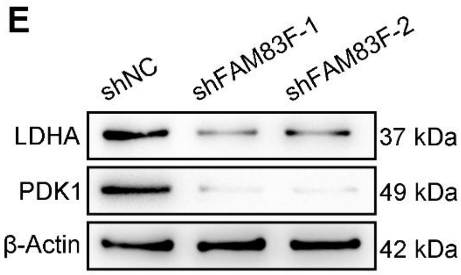

LDHA 37kDa

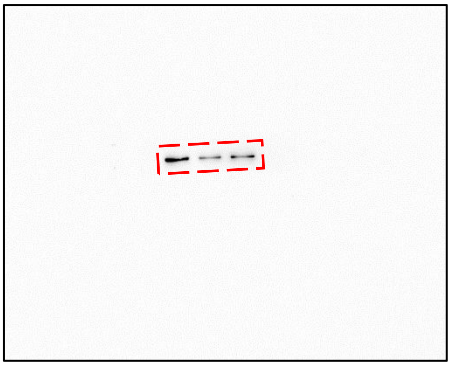

PDK1 49kDa

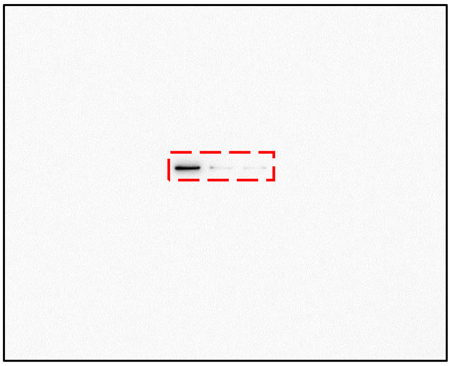

β-Actin 42kDa

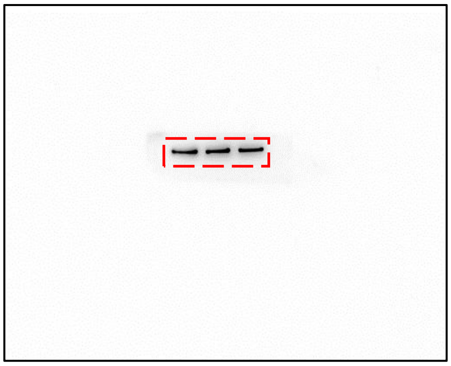

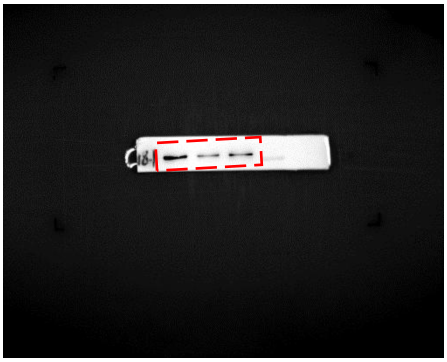

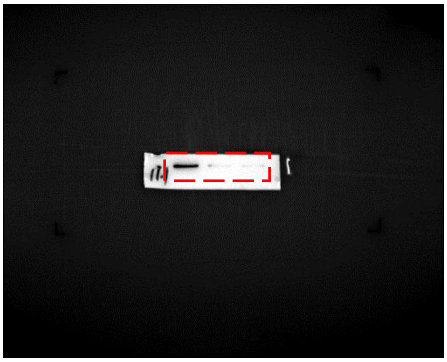

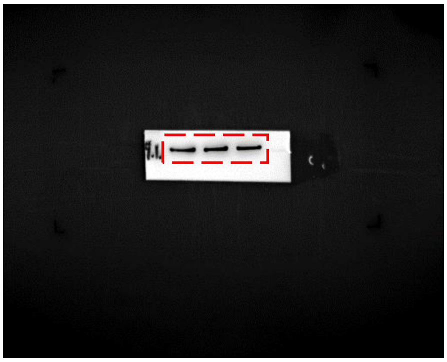

Figure 3F

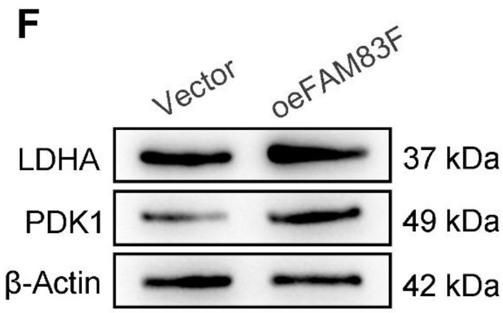

LDHA 37kDa | PDK1 49kDa | β-Actin 42kDa

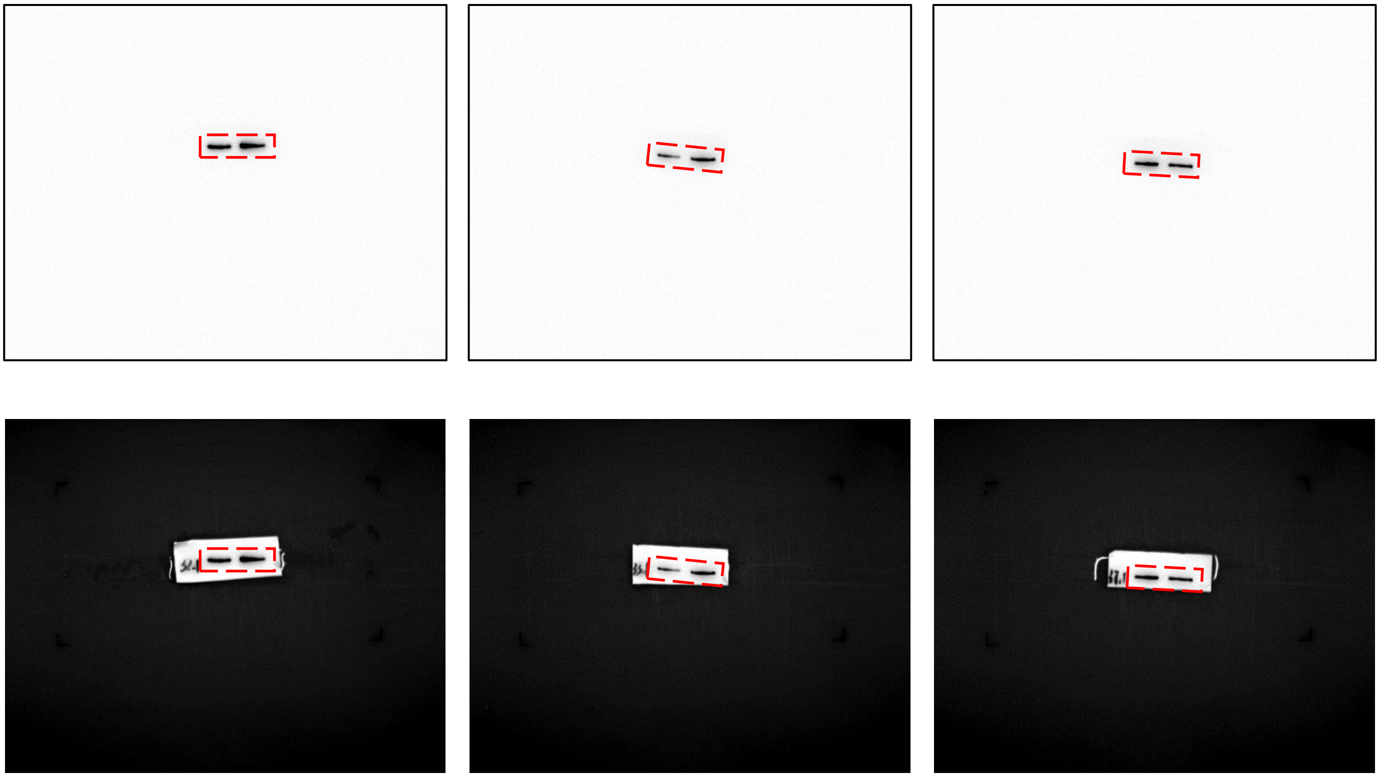

Figure 4B

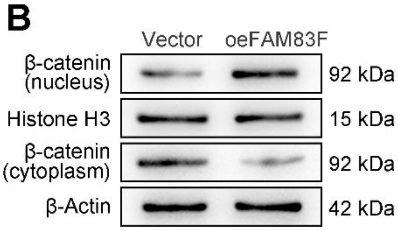

β-catenin (nucleus) 92kDa

Histone H3 15kDa

β-catenin (cytoplasm) 92kDa

β-Actin 42kDa

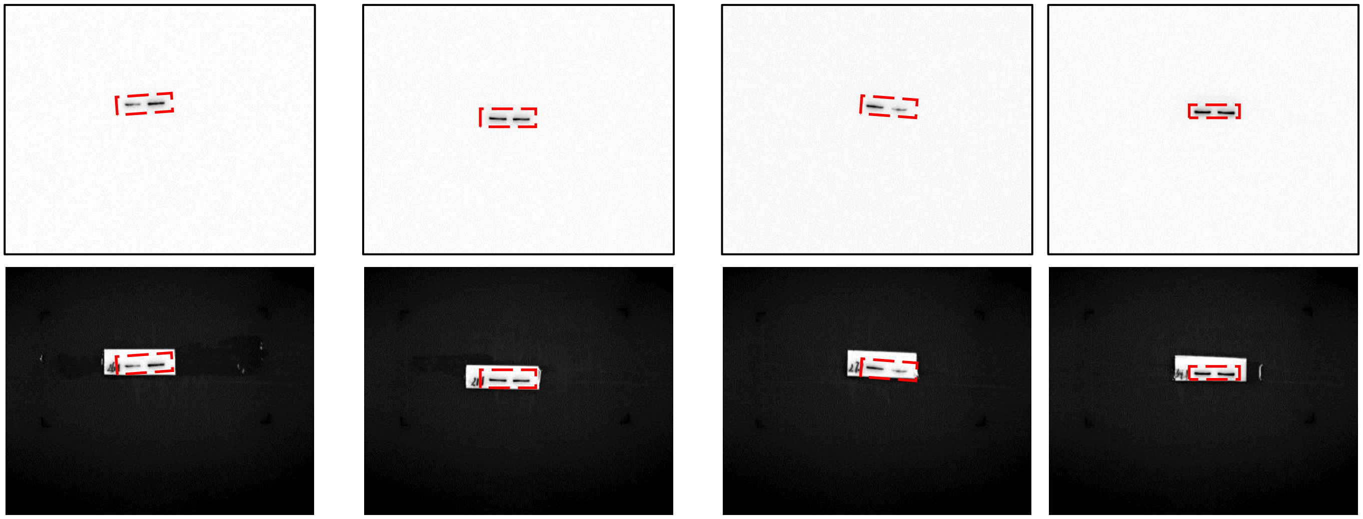

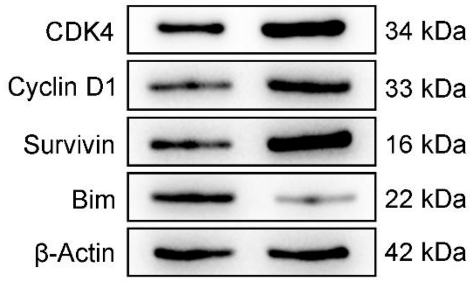

CDK4 34 kDa

Cyclin D1 33 kDa

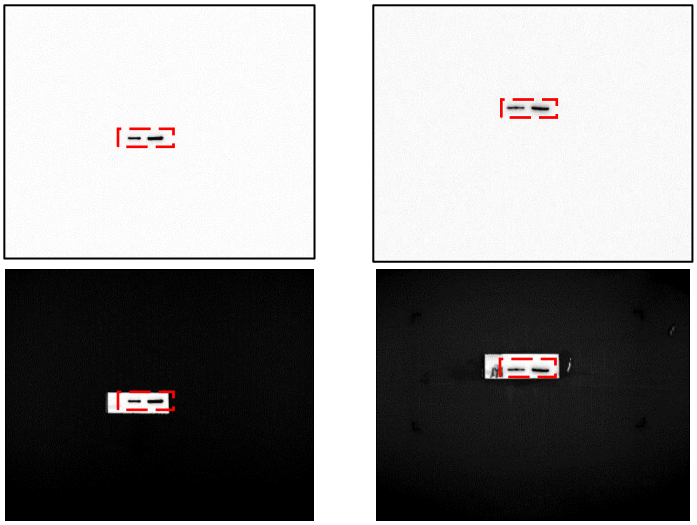

Survivin 16kDa

Bim 22kDa

β-Actin 42kDa

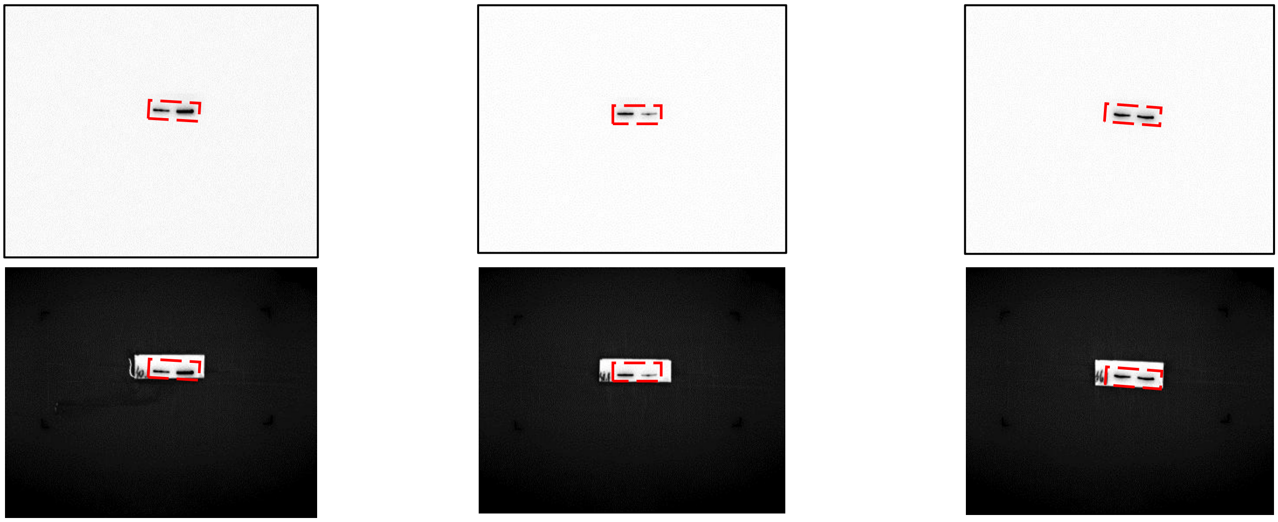

Figure 4C

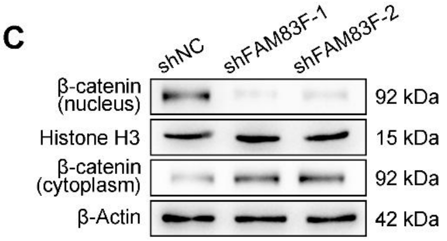

β-catenin (nucleus) 92kDa

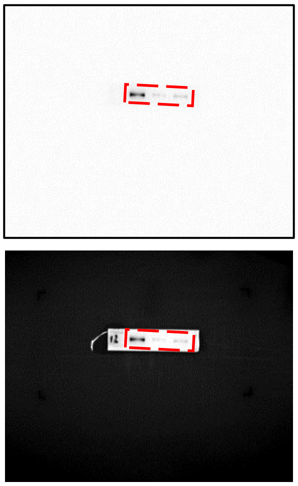

Histone H3 15kDa

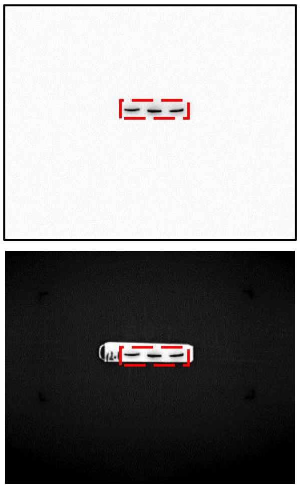

β-catenin (cytoplasm) 92kDa

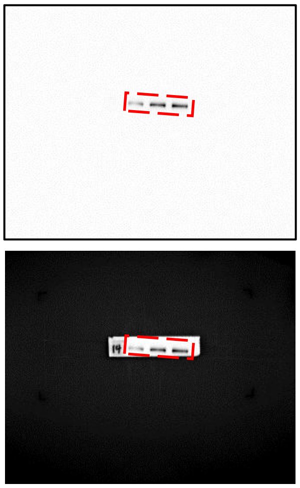

β-Actin 42kDa

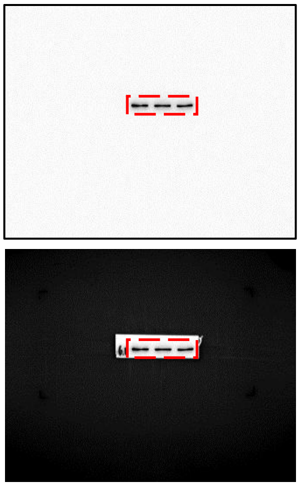

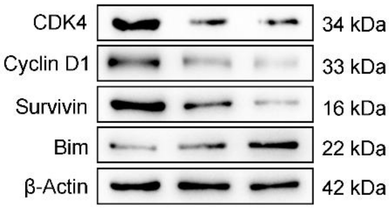

CDK4 34 kDa

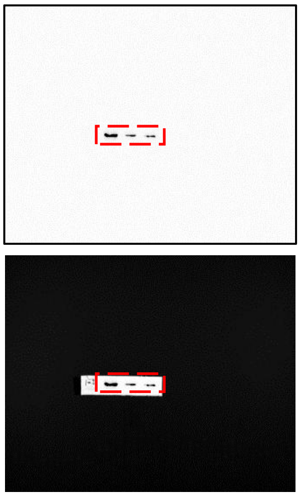

Cyclin D1 33 kDa

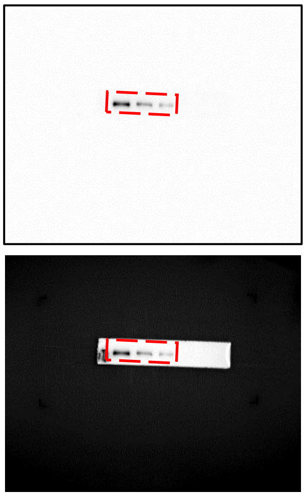

Survivin 16kDa

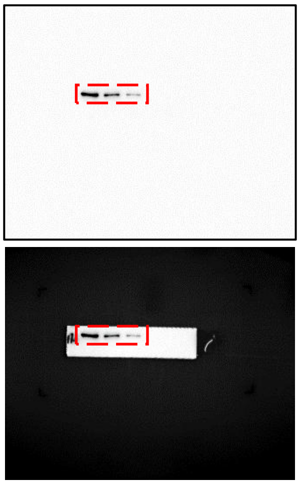

Bim 22kDa

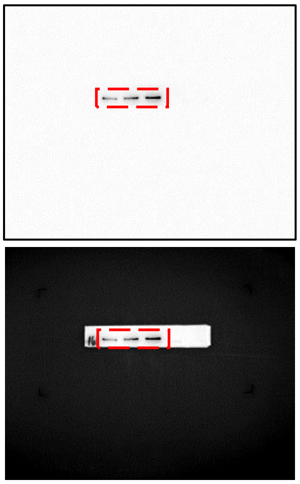

β-Actin 42kDa

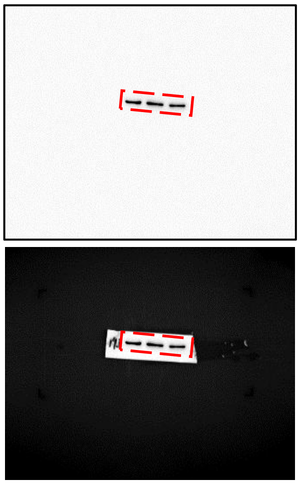

Figure 5E

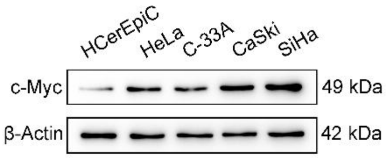

c-Myc 49kDa

β-Actin 42kDa

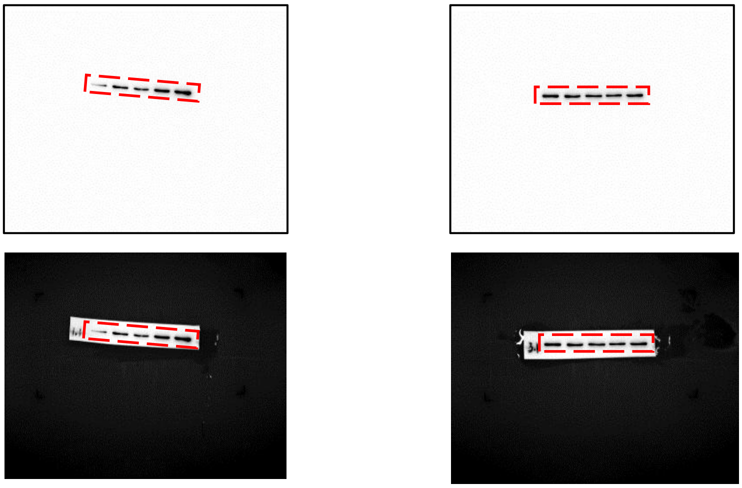

Figure 5G

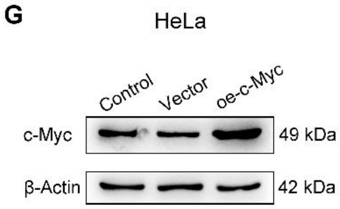

c-Myc 49kDa

β-Actin 42kDa

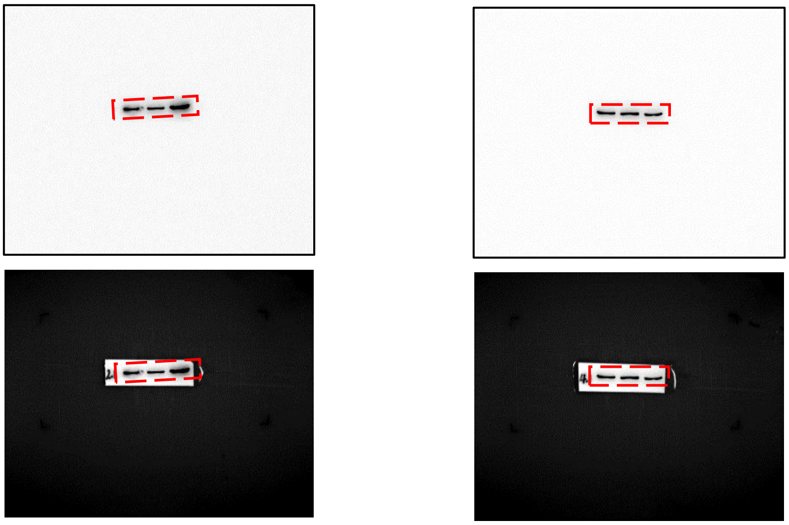

FAM83F 39kDa

β-Actin 42kDa

Figure 5I

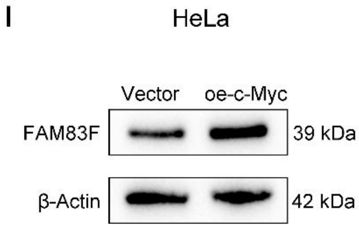

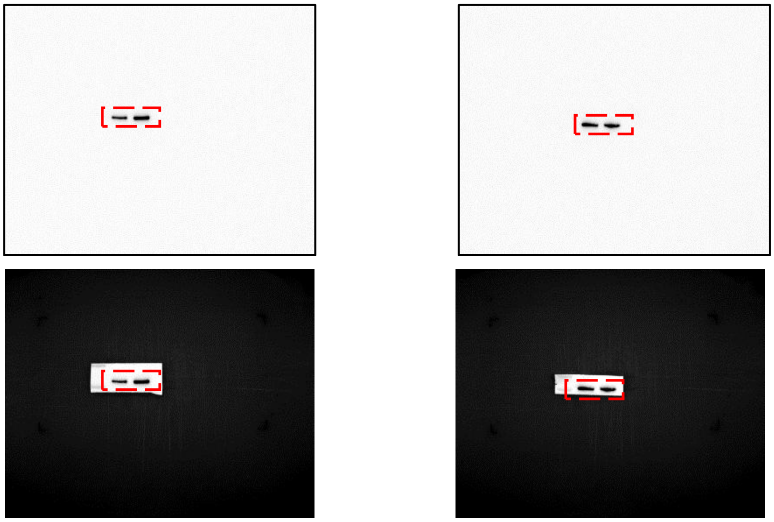

Figure 6C

C

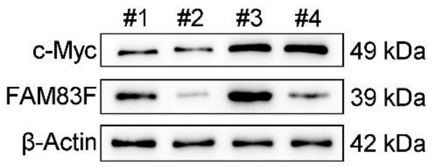

c-Myc 49kDa FAM83F 39kDa β-Actin 42kDa

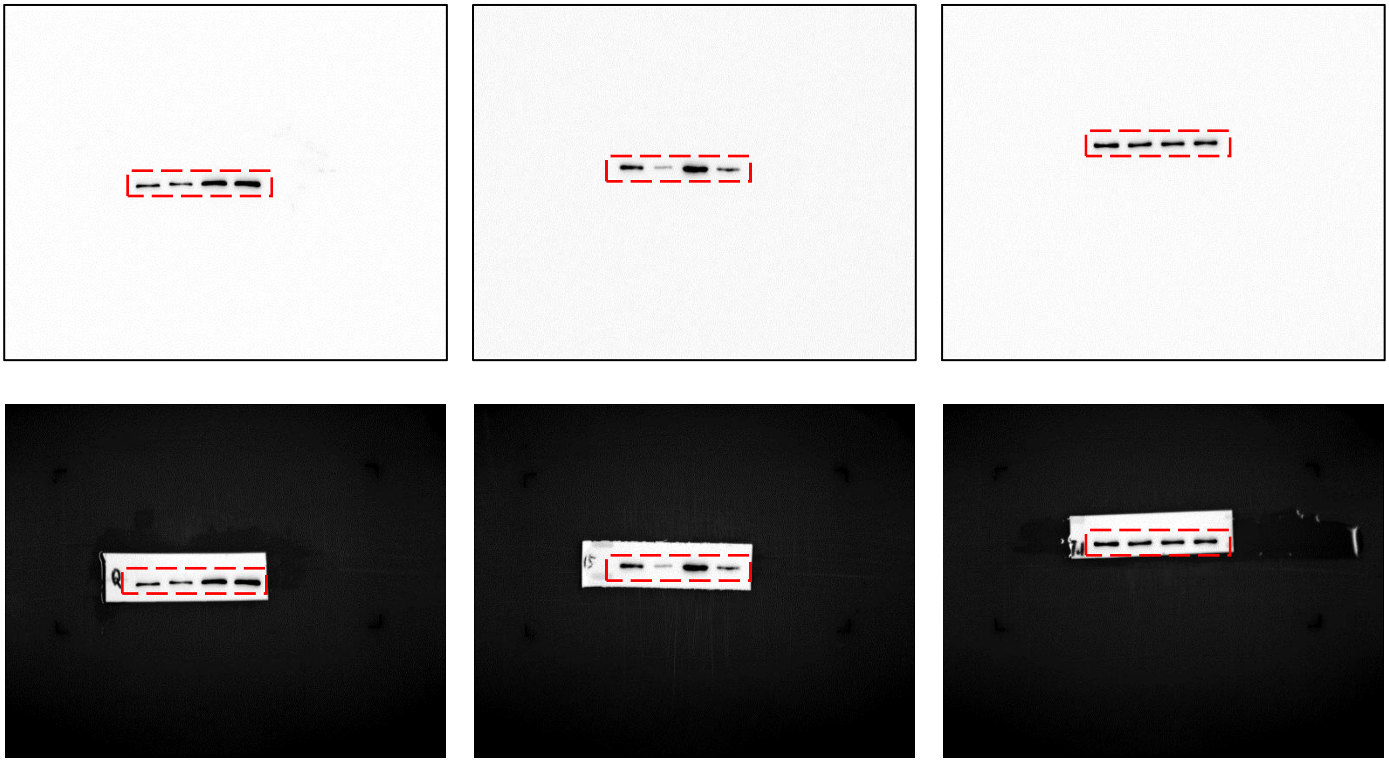

Figure 7D

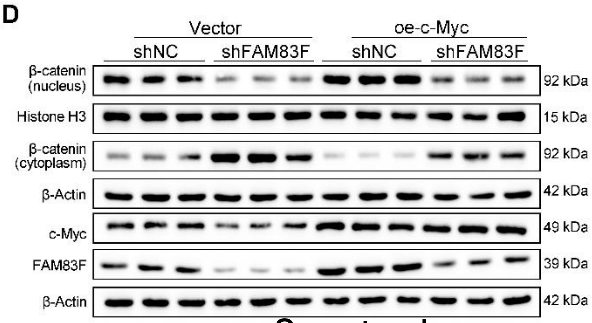

β-catenin (nucleus) 92kDa

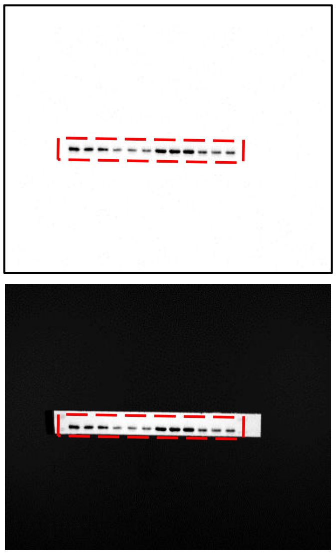

Histone H3 15kDa

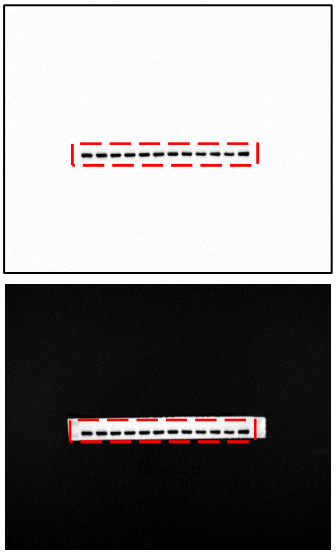

β-catenin (cytoplasm) 92kDa

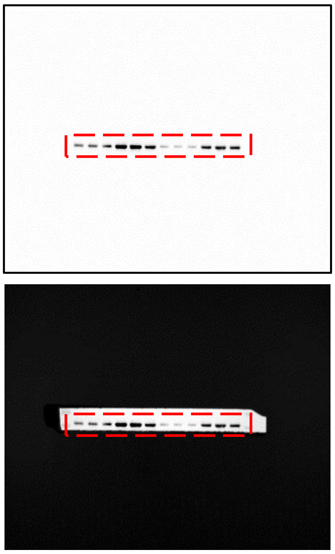

β-Actin 42kDa

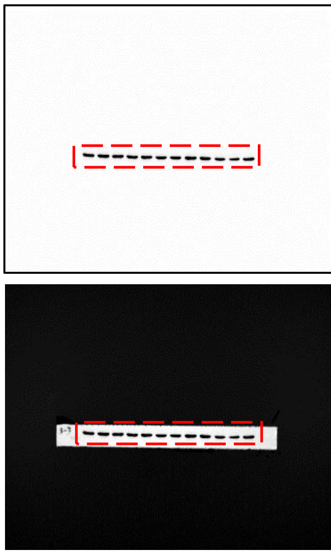

c-Myc 49kDa

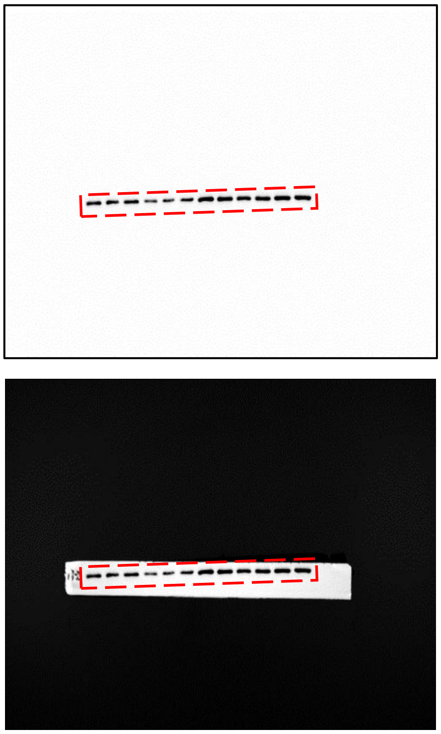

FAM83F 39kDa

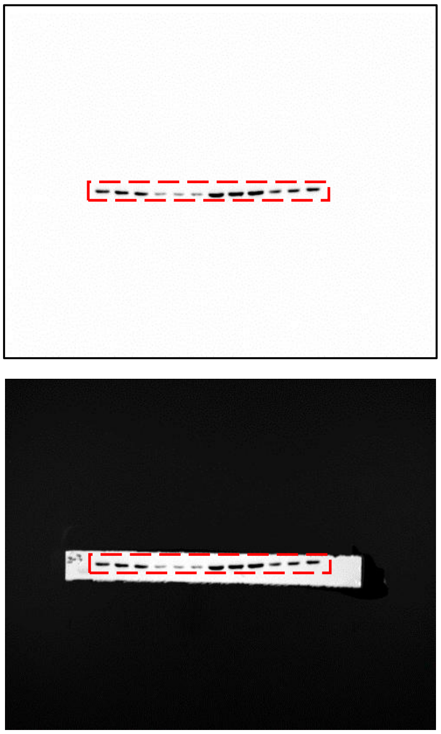

β-Actin 42kDa

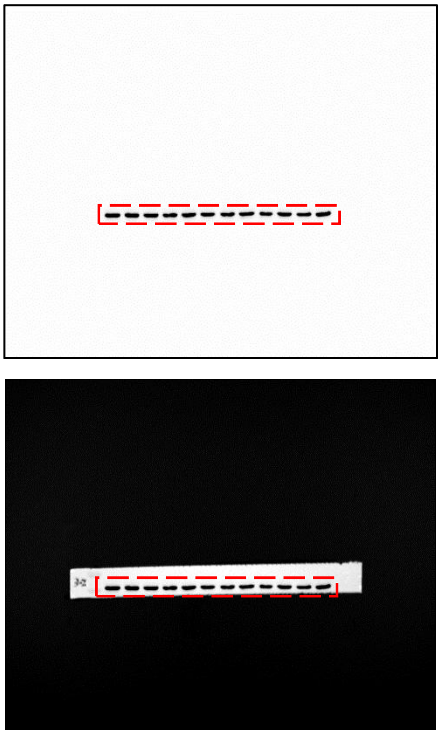

Supplement: Supplementary file 6 — Original Data File [file 41419_2023_6377_MOESM6_ESM.pdf]
